# Theoretical Investigation of Transient Species Following Photodissociation of Ironpentacarbonyl in Ethanol Solution

**DOI:** 10.1021/acs.inorgchem.4c01100

**Published:** 2024-05-28

**Authors:** Michael R. Coates, Ambar Banerjee, Raphael M. Jay, Philippe Wernet, Michael Odelius

**Affiliations:** † Department of Physics, Stockholm University, AlbaNova University Center, SE-106 91 Stockholm, Sweden; ‡ Department of Physics and Astronomy, Uppsala University, P.O. Box 516, SE-751 20 Uppsala, Sweden

## Abstract

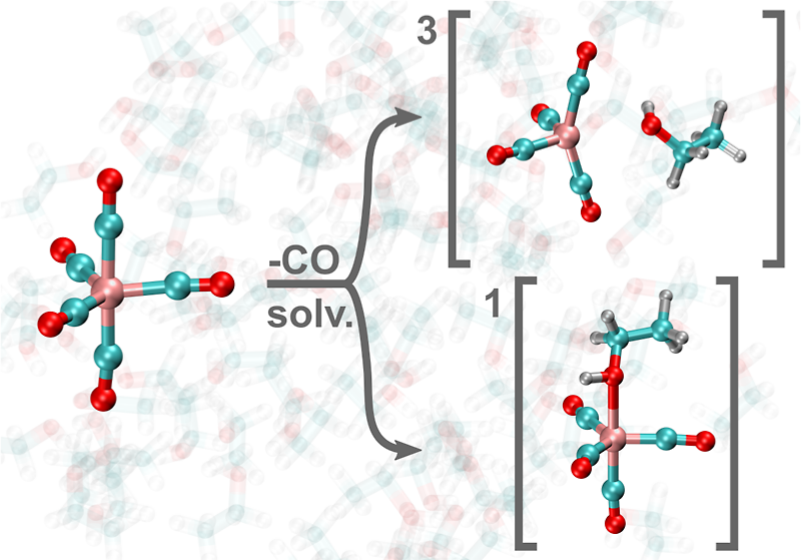

Photodissociation of ironpentacarbonyl [^1^Fe(CO)_5_] in solution generates transient species in different electronic states, which we studied theoretically. From ab initio molecular dynamics simulations in ethanol solution, the closed-shell parent compound ^1^Fe(CO)_5_ is found to interact weakly with the solvent, whereas the irontetracarbonyl [Fe(CO)_4_] species, formed after photodissociation, has a strongly spin-dependent behavior. It coordinates a solvent molecule tightly in the singlet state [^1^Fe(CO)_4_] and weakly in the triplet state [^3^Fe(CO)_4_]. From the simulations, we have gained insights into intersystem crossing in solvated irontetracarbonyl based on the distinct structural differences induced by the change in multiplicity. Alternative forms of coordination between ^1^Fe(CO)_4_ and functional groups of the ethanol molecule are simulated, and a quantum chemical investigation of the energy landscape for the coordinated irontetracarbonyl gives information about the interconversion of different transient species in solution. Furthermore, insights from the simulations, in which we find evidence of a solvent exchange mechanism, challenge the previously proposed mechanism of chain walking for under-coordinated metal carbonyls in solution.

## Abstract 2

^1^Fe(CO)_5_ and the photoproducts ^1^Fe(CO)_4_ and ^3^Fe(CO)_4_ have been studied by ab initio simulation and quantum chemistry calculations. In ethanol solution, ^1^Fe(CO)_5_ is found to be unreactive toward the solvent, while ^1^Fe(CO)_4_ tightly binds a solvent molecule via the hydroxyl (−OH) and alkyl (−CH) groups and ^3^Fe(CO)_4_ weakly coordinates the solvent through electrostatic interactions.

## 1 Introduction

Transition-metal carbonyls have played a significant role in the understanding of the photochemistry of organometallic complexes.^1,2^ The ultraviolet excitation of these metal complexes can result in the loss of one or more carbonyls, which creates transient intermediates capable of interacting and reacting with the available solvent. The initial photoproduct dynamics of these transient intermediates is analogous to the reactive sites of metalloenzymes (for example: hemoglobin^3^ or cytochrome P450^4^), which bind and/or react with the aqueous environment, often accompanied by structural and electronic changes. With respect to photoinitiated dynamics, the loss of a ligand may include changes in multiplicity that give subpicosecond intersystem crossings occurring well before vibrational cooling of the reactive metal complex.^5^ Studies of the photoinitiated dynamics of metal carbonyls have provided rich insights into solvent coordination, reactivity of intermediates, the energies of transition states, and spin–orbit coupled interactions, which are important properties determining catalytic processes. In this light, ironpentacarbonyl [^1^Fe(CO)_5_] has served as a basis for many studies as a benchmark metal carbonyl exhibiting the aforementioned reactivity and dynamics.

In the gas and liquid phases, the excitation of ^1^Fe(CO)_5_ to a bright metal-to-ligand charge-transfer state gives prompt dissociation of a carbonyl ligand following ultrafast internal conversion to a dissociative metal-centered (MC) state. With an electronic structure obeying the 18-electron rule,^5^ the ^1^Fe(CO)_5_ complex is stable, while the undercoordinated 16-electron ^1^Fe(CO)_4_ complex is unstable. The production of ^1^Fe(CO)_4_ leads to ligand addition of an available solvent molecule in many solvents, namely, alkane,^6,7^ alcohol,^8,9^ and alkylsilanes.^10^ Pump–probe IR studies of Fe(CO)_5_ in methanol, 1-butanol, 1-hexanol, and *tert*-butyl alcohol indicate that dynamics in the singlet manifold is rapidly involving coordination of alcohol from the hydroxyl (−OH) group.^8^ Additionally, pump–probe X-ray absorption fine structure (XANES) measurements of ^1^Fe(CO)_5_ in ethanol^9^ confirm the coordination of ethanol via the hydroxyl group. The authors use B3LYP/6-31G** optimized structures to calculate theoretical XANES spectra and show that the coordination of ethanol to ^1^Fe(CO)_4_ begins around 3 ps, on the time scale where vibrational cooling is dominant. Complicating this understanding is a complementary study in heptane that indicates that singlet C–H coordination arises via a triplet ironpentacarbonyl ^3^Fe(CO)_4_ intermediate, which forms within 33 ps and decays up to 600 ps, concurrent with the formation of the singlet ^1^Fe(CO)_4_-solvent product.^10^ In alcohol solution, this triplet ^3^Fe(CO)_4_ lifetime varies with alcohol chain length between 40 and 300 ps, resulting in the formation of the hydroxyl coordinated ^1^Fe(CO)_4_.^10^ Elucidation of the initial transient species by femtosecond Fe L-edge (2p–3d) resonant inelastic X-ray scattering (RIXS) indicates that the ligation of ethanol to ^1^Fe(CO)_4_ occurs within 200–300 fs, and although modeling of the RIXS probe could strictly not distinguish between solvent-coordination or geminate recombination, it was suggested to be a favorable reaction on the basis of a vibrationally hot intermediate reacting with the available solvent in the first solvation shell.^11^

The interconversion between hydroxyl (−OH) and alkyl (−CH) coordination in a metal complex has been explained in terms of a “chain-walking” mechanism,^12^ whereby a rearrangement occurs within the complex with the coordinating solvent molecule. To date, this mechanism in relation to the coordination of a transient species has been discussed for Cr(CO)_6_,^13^ where the attachment of a short chain alcohol occurs via the alkyl groups first, followed by successive bonding rearrangements along the alkyl chain until the energetically favored hydroxyl coordination occurs. By means of a model, the authors find a competition between a “chain-walking” mechanism and an intermolecular rearrangement occurring via the loss of an alkyl coordinated ligand and formation of a hydroxyl coordinated ligand. This mechanism has also been discussed in terms of Mn(ppy)(CO)_4_ (ppy = 2-phenylpyridine),^14^ where pump–probe IR measurements in tetrahydrofuran, 1,4-dioxane, *n*-Bu_2_O, and dimethyl sulfoxide (DMSO) show that the solvent molecules coordinate to Mn(ppy)(CO)_3_ via the alkyl bonds first, followed by interconversion to the stable oxygen bound configurations at longer times. The authors propose both intra- and intermolecular mechanisms, highlighting the difficulty in finding an experimental distinction between the two mechanisms.

The theoretical understanding of the coordination in the singlet and triplet states of irontetracarbonyl is limited to static structural models. To date, many computational studies have provided benchmark energies of ^1^Fe(CO)_5_, ^3^Fe(CO)_4_ and ^1^Fe(CO)_4_ in the gas phase. In particular, the minimum energy crossing point (MECP) between the ground state singlet and triplet potential energy surfaces of Fe(CO)_4_ has been explored by coupled cluster singles and doubles and perturbatively triples CCSD(T) calculations.^15^ The authors found that with an extrapolation to the basis set limit and by including scalar relativistic correlation effects, an observed singlet–triplet energy gap of only 2 kcal mol^–1^ was observed. In a similar study^6^ using density functional theory (DFT), the energies of the singlet and triplet ground states of Fe(CO)_4_ and the MECP were calculated at the B3PW91**/TZV level of theory. They determined a comparably small singlet–triplet gap of 3.5 kcal mol^–1^ evaluated at the triplet ground state minimum. Snee et al.^10^ also calculated the energies of the singlet and triplet ground states of Fe(CO)_4_ at the B3LYP/6-31G** level of theory and found a small singlet–triplet gap of 5.43 kcal mol^–1^ evaluated at the triplet ground state minimum. In a follow-up study,^8^ the authors considered a methanol ligated Fe(CO)_4_–(CH_3_OH) species using the same functional and basis set. They found the existence of a shallow minimum in the triplet state corresponding to an Fe–O bond length of 2.92 Å and an associated MECP at an Fe–O bond length of 2.85 Å, indicating a lack of direct coordination to the metal center. Their DFT calculations indicated a weak triplet interaction energy (Δ*E*_int_) of −3.02 kcal mol^–1^ and an MECP barrier of −3.01 kcal mol^–1^, where at an Fe–O bond length of 2.85 Å the triplet state is lower in energy than the corresponding singlet state. At this MECP, they find a calculated spin–orbit coupling (SOC) of 2.6 cm^–1^. The authors concluded based on the small SOC that an ISC to the ligated Fe(CO)_4_–OH would occur due to a small barrier height, rather than via the SOC. This is rationalized in terms of a coordinated singlet Fe–O bond length being roughly 1 Å shorter than the shallow minimum in the triplet state at 2.92 Å. The authors state that only a small rearrangement of the molecules is necessary to overcome the small barrier and form the ligated singlet Fe(CO)_4_–OH.

These previous studies indicate the need for a deeper understanding of the interconversion of transient species resulting from the initial photodissociation of ^1^Fe(CO)_5_. This motivates the following study of the complex reactive landscape by theoretical simulation. Ethanol solutions of ^1^Fe(CO)_5_, ^1^Fe(CO)_4_, and ^3^Fe(CO)_4_ were simulated using ab initio molecular dynamics (AIMD) to assess the solvation and fluctuation of the parent compound and thermally equilibrated transient species. The intersection of the lowest triplet and singlet states of the Fe(CO)_4_–EtOH complex is explored in search for the mechanism of intersystem crossing. We then present characterization of the minima and transition states of different coordination conformations for ^1^Fe(CO)_4_–EtOH in the singlet state. We derive insights into the chemical bonding by breaking down the molecular orbitals into atomic contributions via a projected density of states (PDOS) analysis of the Kohn–Sham orbitals. The AIMD simulation trajectories also give a new perspective on reaction pathways for interchange and evolution of different species, which can be contrasted against the previously proposed model of chain-walking.^12^

## 2 Methods

### 2.1 AIMD Simulations

The theoretical investigation of the solvation of ^1^Fe(CO)_5_, ^1^Fe(CO)_4_, and ^3^Fe(CO)_4_ in ethanol was based on Born–Oppenheimer AIMD simulations. The AIMD simulations of the periodic models were performed using DFT with a mixed Gaussian and plane wave method in the CP2K set of programs^16^ by using the BLYP functional^17,18^ with Grimme’s D3 van der Waals correction^19,20^ in a cubic cell (*a* = 21.6579 Å) and *a* plane wave cutoff of 300 Ry in the description of the electron density. The GTH pseudopotentials^21^ were used (with explicit inclusion of semicore 3s3p electrons for Fe) together with the GTH-DZVP (Fe) and GTH-TZVP (C, O, H) basis sets. The systems contained 100 ethanol molecules and were initialized from the previous Car–Parrinello MD simulation^11^ at a density of 0.785 g/cm^3^. The simulations of Fe(CO)_4_ (in the singlet and triplet spin states) started from the same configuration after removing a CO from the Fe(CO)_5_ complex and hence have a slightly lower density. The simulations were run at 300 K in the *NVT* ensemble with a velocity rescaling thermostat (CSVR).^22^ Each simulation was equilibrated for at least 10 ps and subsequently allowed to evolve for a production run of slightly more than 50 ps. To investigate the possibility of alkyl coordination, ^1^Fe(CO)_4_ AIMD simulations corresponding to coordination via the −HC_α_ (−CH_2_OH) and −HC_β_ (−CH_3_) sites on the ethanol molecule were initiated from the hydroxyl coordinated ^1^Fe(CO)_4_ AIMD trajectory. To do so, a specific Fe–H_C_ distance was restrained to a harmonic potential of 3.0 Å with an associated spring constant of 0.005 hartree/bohr^2^. For the AIMD simulation with −HC_α_ coordination, the restraint was applied for 21.5 ps, followed by an unrestrained sampling for 15 ps. For the AIMD simulation with −HC_β_ coordination, the restraint was applied for 23.5 ps followed by an unrestrained sampling for 15 ps. The analysis of the alkyl coordinated trajectories contains results only from the unrestrained AIMD simulations in both coordination sites. For structural analysis, radial distribution functions, denoted *g*(*r*), were computed for all iron–carbonyl distances (Fe–C and Fe–O) and for all iron–ethanol distances (Fe–O_H_, Fe–C_H_, Fe–H_O,_ and Fe–H_C_).

### 2.2 Quantum Chemistry Calculations

To assess the various local minima and saddle points in the potential energy surface of the ethanol coordinated ^1^Fe(CO)_4_ and to relate to weaker solvent interactions for ^1^Fe(CO)_5_ and ^3^Fe(CO)_4_, a library of structures were formed by optimizing structures in DFT using the TPSSh functional^23,24^ with the def2-TZVP basis set^25,26^ with implicit solvation given by the conductor-like polarizable continuum (CPCM) model^27^ using ethanol as a solvent. All subsequent cluster calculations include implicit CPCM solvation unless stated otherwise. The possible coordination between the hydroxyl (−OH), alkyl (−HC_α_ or −HC_β_) in either an axial or equatorial coordination to ^1^Fe(CO)_4_ leads to six minima and their corresponding transition states. All optimizations of both the minima and transition states were completed using the Gaussian 16 quantum chemistry suite.^28^ For each of the TPSSh/def2-TZVP optimized minima and transition state structures, we assess the BLYP functional used in the AIMD simulations by calculating single point energies in CP2K with the BLYP functional without CPCM. The calculations with the B3LYP hybrid functional in CP2K were performed with the same pseudopotentials as the BLYP calculations and with Grimme’s D3 van der Waals for the B3LYP functional. For the B3LYP calculations, the Hartree–Fock (HF) exchange was approximated by the auxiliary density matrix method^29^ in which the exact HF exchange integrals are evaluated using a smaller auxiliary atomic basis set. Finally, we make a rigorous assessment of the DFT energies by calculating CCSD(T) energies in the domain-based local pair natural orbital (DLPNO) approach^30−32^ to accelerate the computational time of the calculations. Scalar relativistic effects in the DLPNO–CCSD(T) calculations were included by the zeroth order regular approximation (ZORA) method^33^ using the ZORA-def2-TZVP basis set.^34^ The TPSSh and DLPNO–CCSD(T) calculations were performed using the ORCA quantum chemistry package, version 5.0.3.^35^ Implicit solvation was included in all DLPNO–CCSD(T) calculations with CPCM unless stated otherwise.

Additional supporting calculations were performed in ORCA using the n-electron valence state perturbation theory (NEVPT2) level of theory^36,37^ based on a complete active space self-consistent field (CASSCF) wave function. To construct the CASSCF wave function, we account for 10 electrons in 10 orbitals, denoted CAS(10e, 10o), in an active space, which contains the occupied d−σ_co_, d_*xz*_, d_*yz*_, d_*xy*_,  and the unoccupied d_*z*^2^_–σ_CO_^*^, d_*xz*_–π_CO_^*^, d_*yz*_–π_CO_^*^, d_*xy*_–π_CO_^*^, –π_CO_^*^ orbitals as shown in Figure S1. With the removal of one CO ligand and replacement by an ethanol ligand, we use the same active space and note that the lowest unoccupied molecular orbital (LUMO) changes character to form an antibonding orbital with the ethanol highest unoccupied molecular orbital (HOMO). To draw a connection between the singlet and triplet ground states of Fe(CO)_4_, we construct a rigid 2-dimensional scan of the θ_C–Fe–C_ angle and the Fe–O_H_ distance by taking the optimized geometry of ^1^Fe(CO)_4_–OH EQ from the structure library and recalculating the points along the scan by NEVPT2 based on a SA(1S + 1T)-CAS(10e, 10o)/def2-TZVP wave function with the active space defined above. Starting with the ^1^Fe(CO)_4_–OH EQ structure, the Fe–O distance varied between 1.5 and 3.5 Å, while the axial C–Fe–C angle was varied from 150 to 190° to bring the cluster close to the optimized ^3^Fe(CO)_4_···OH structure (Fe–O = 3.18 Å and θ_C–Fe–C_ = 150.6°).

#### 2.2.1 Electronic DOS

In addition to the energetic investigation outlined above, the electronic structures of the bonding arrangements were analyzed by calculations in CP2K of the PDOS corresponding to atomic orbital contributions of each atom type to the occupied and unoccupied Kohn–Sham orbitals. Furthermore, PDOS values were calculated for specific atoms that were involved in coordination to the iron center. All curves were obtained using the same computational framework as the AIMD simulations but applied to the structure library optimized at the TPSSh/def2-TZVP + CPCM(ethanol) level of theory (see Table 1). In an effort to connect the molecular orbital characters between all calculated geometries, we note the orbital character (Fe 3d_*xz*_, 3d_*yz*_, 3d_*xy*_, , 3d_*z*^2^_, and CO σ π_*x*_, π_*y*_, π_x_^*^, π_y_^*^) and write the irreducible representation labels in the *D*_3h_ point group of ^1^Fe(CO)_5_ in parentheses. In addition, we follow the axis labels set by Hoffmann and co-workers,^38^ which takes the *z*-axis as vertical, *x*-axis as horizontal, and the *y*-axis as out of the page. In their study, the authors extensively detailed the orbital energies and symmetries corresponding to ^1^Fe(CO)_5_, and axially or equatorially uncoordinated ^1^Fe(CO)_4_ AX and ^1^Fe(CO)_4_ EQ, to which this study makes significant reference in the analysis of PDOS. To make a connection from the implicitly solvated structure library to the periodic AIMD simulation, a snapshot of the simulation was sampled every 100 fs to give 506, 510, and 526 snapshots for ^1^Fe(CO)_5_, ^1^Fe(CO)_4_, and ^3^Fe(CO)_4_ simulations, respectively. All PDOS were convoluted with a Gaussian function using a full width at half-maximum of 0.02 eV.

#### 2.2.2 Local Energy Decomposition

In order to characterize the interactions between Fe(CO)_4_ and ethanol solvent molecules, we performed an LED analysis of the DLPNO-CCSD(T) energy for selected complexes. In the LED method, formulated by Bistoni et al.,^39^ the total energy (Δ*E*) in eq 1 decomposed into terms, which are further broken down into contributions from HF and CCSD(T)
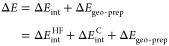
1

The first term Δ*E*_int_, the interaction energy, is decomposed into the energies coming from HF (Δ*E*_int_^HF^) and the dynamical electron correlation coming from CCSD(T) (Δ*E*_int_^C^). The final term, Δ*E*_geo-prep_, is the energy associated with the geometrical distortion (so-called deformation energy) in taking the isolated monomers from their optimized geometries into the geometries of the fragments within the complex.

The Δ*E*_int_^HF^ term can be further decomposed as follows
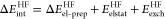
2

The Δ*E*_el-prep_^HF^ is a sum of the electronic energetic cost to bring the isolated electronic structures of the fragments into the interaction within the complex. The final two terms represent the electrostatic (Δ*E*_elstat_^HF^) and exchange (Δ*E*_exchange_^HF^) interaction energies of the fragments in the complex.

The correlation energy term (Δ*E*_int_^C^) introduced by the CCSD(T) calculation is divided into three terms
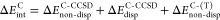
3

These are the nondispersive (Δ*E*_non-disp_^C-CCSD^) charge transfer interactions, the London dispersion interaction energy (Δ*E*_disp_^C-CCSD^), and the CCSD(T) triples correction (Δ*E*_int_^C-(T)^) to the interaction energy. The energy decomposition in eqs 1–3 allows us to distinguish the main character of the interactions for different species.

## 3 Results

In Section 3.1, AIMD simulations are compared to highlight differences in interactions of the ^1^Fe(CO)_5_ reactant and transient Fe(CO)_4_ species in the singlet and triplet states. In the simulations of solvated ^1^Fe(CO)_4_ and ^3^Fe(CO)_4_ complexes, iron preferentially coordinates the solvent ethanol via the hydroxyl group. The reaction coordinate for intersystem crossing is investigated by potential energy scans based on the optimized singlet and triplet Fe(CO)_4_–EtOH geometries. We use the optimized gas phase complexes (^1^Fe(CO)_5_, ^1^Fe(CO)_4_, and ^3^Fe(CO)_4_) and the corresponding simulation trajectories to derive molecular orbital insights via PDOS calculations. In Section 3.2, similar simulations and analyses are applied to the different coordinations (^3^Fe(CO)_4_···OH, ^1^Fe(CO)_4_–HC_α_, and ^1^Fe(CO)_4_–HC_β_) and transition states determined with a saddle-point search of the singlet ^1^Fe(CO)_4_ transient species with an ethanol ligand. Finally, in Section 3.3, quantum chemistry calculations and energy decomposition are performed to understand the orbital interaction that contributes to the bonding in ^1^Fe(CO)_4_–OH, ^1^Fe(CO)_4_–HC_α_, and ^3^Fe(CO)_4_···OH.

### 3.1 Reactivity of Singlet and Triplet Irontetracarbonyl

**Figure 1:**
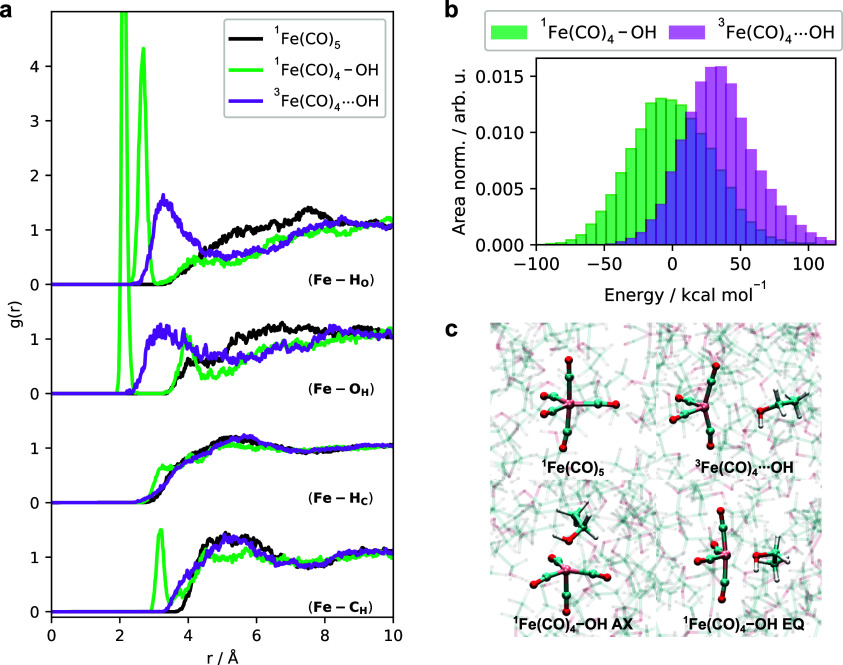
(a) Radial distribution functions *g*(*r*) sampled from the ^1^Fe(CO)_5_, ^1^Fe(CO)_4_–OH, and ^3^Fe(CO)_4_ simulations. The coordination around the iron complexes are seen in *g*(*r*) for Fe–H_O_, Fe–O_H_, Fe–H_C_, and Fe–C_H_. Results for ^1^Fe(CO)_5_ are colored black, for ^1^Fe(CO)_4_–OH are colored green, and for ^3^Fe(CO)_4_ are colored purple. For clarity, the individual *g*(*r*) classes are plotted separately in Figure S2. (b) Distribution of the potential energies from the ^1^Fe(CO)_4_–OH and ^3^Fe(CO)_4_ simulations, following the same color scheme. Both energy distributions are area normalized and the mean of the singlet trajectory is used as reference energy. (c) Representative snapshots from each AIMD simulation, from which two distinct configurations are observed in the ^1^Fe(CO)_4_–OH simulation.

The innate differences in bonding and solvent coordination based on AIMD simulations of ethanol solutions of ^1^Fe(CO)_5_, ^1^Fe(CO)_4_, and ^3^Fe(CO)_4_ are shown in Figure 1. For each AIMD simulation, we characterize the coordination of the iron carbonyl complex with radial distribution functions, *g*(*r*), between the iron atom and the atoms in the ethanol molecules. From this, we obtain *g*(*r*) corresponding to the –OH hydrogen (Fe–H_O_), the –OH oxygen (Fe–O_H_), –CH hydrogen (Fe–H_C_), and the –CH carbon (Fe–C_H_) atoms. The reactant ^1^Fe(CO)_5_ (shown in black in Figure 1a) is very weakly coordinated, with all *g*(*r*) indicating a broad first solvation shell beginning at roughly 4 Å. This is an agreement with the results of previous classical and Car–Parrinello MD simulations.^11^ In comparison, the transient species ^1^Fe(CO)_4_–OH (shown in green) and ^3^Fe(CO)_4_ (shown in purple) in Figure 1a provide two clear results: ^1^Fe(CO)_4_ is strongly coordinated with the solvent, forming a stable ^1^Fe(CO)_4_–OH complex, while ^3^Fe(CO)_4_ is loosely coordinated, albeit with a distinctly ordered first solvation shell. The transient species ^1^Fe(CO)_4_ shows a sharp peak centered at 2.1 Å in the Fe–O_H_*g*(*r*), and a less sharp peak centered at 2.7 Å in the Fe–H_O_*g*(*r*). These features correspond to the preferential coordination of one ethanol solvent to the undercoordinated ^1^Fe(CO)_4_ via the hydroxyl group, which we find to be largely in the axial coordination, denoted ^1^Fe(CO)_4_–OH AX as opposed to equatorial coordination, denoted ^1^Fe(CO)_4_–OH EQ. The *g*(*r*) for the Fe–H_O_ distance is clearly longer than the Fe–O_H_ distance, indicating the coordination of the –OH oxygen to the iron center. Additionally, there is a sharp peak, centered at 3.2 Å, appearing in the Fe–C_H_*g*(*r*), which is attributed to the α-carbon corresponding to the ethanol ligand bonded via the –OH oxygen. The transient species ^3^Fe(CO)_4_ in the triplet state shows a weak hydroxyl coordination in the first solvation shell at Fe–O_H_ distances around 3.0 Å, which is an intermediate distance between the coordinating Fe–O_H_ distance and first solvation shell distance in ^1^Fe(CO)_4_, but still more pronounced that in the case of ^1^Fe(CO)_5_.

We notice based on the Fe–H_O_ and Fe–O_H_*g*(*r*) in Figure 1a for the ^1^Fe(CO)_4_–OH and ^3^Fe(CO)_4_ simulations that the distributions are partially overlapping in the bonding region below 3 Å; however, as it will be described in Section 3.3, the triplet state is fundamentally unreactive and the interaction with the solvent is weak. The triplet ground state is described by an electron configuration that fills all d-orbitals, negating the possibility of coordination with an electron donating ligand. We explore in detail in Section 3.3 the preferential association of the hydroxyl group in the triplet state, occurring despite the weak electronic interaction. For both transient Fe(CO)_4_ species, we integrate the Fe–O_H_*g*(*r*) up to the correspond first minimum of ^1^Fe(CO)_4_ and ^3^Fe(CO)_4_ (3.0 and 5.5 Å, respectively). Based on this, it is determined that there is a tightly bound ethanol to ^1^Fe(CO)_4_, coordinating via the hydroxyl group. In ^3^Fe(CO)_4_, the integration shows that there are roughly five weakly bound ethanol solvent molecules and no distinction for the closest coordination is made (described below).

In Figure 1b, the distributions of potential energies for the ^1^Fe(CO)_4_–OH and ^3^Fe(CO)_4_ simulations are displayed, with energies taken with respect to the average energy of the ^1^Fe(CO)_4_–OH distribution. Based on this, we find that the singlet state is favored energetically by roughly 50 kcal mol^–1^. Lastly, in Figure 1c, representative snapshots from each of the three AIMD simulations are shown. For the ^1^Fe(CO)_4_–OH AIMD trajectory, we find that although the majority of the trajectory corresponds to an essentially bipyramidal coordination with a ^1^Fe(CO)_4_–OH AX structure, there exists an exchange of axial and equatorial ligands to give an equatorially coordinated ethanol. In Figure S3 we display the θ_O–Fe–C_ angles that show that the ^1^Fe(CO)_4_–OH EQ species exists for roughly 8 ps during the 51 ps of the dynamics simulation. With respect to the sampled results in Figure S4a,b, both species are contained within the *g*(*r*) and potential energy distributions. Additionally, Figure S4 shows a representative snapshot of the dynamics where the hydrogen bonding network of ethanol solution is perturbed by the weak interaction between ^3^Fe(CO)_4_ and a few nearby solvent molecules. By plotting the Fe–O_H_ distances of the close lying ethanol molecules, we find that one ethanol weakly coordinates the metal center, interchanging with the surrounding hydrogen bonded network. We therefore simplify the picture to consider the preferential association of one ethanol molecule to the ^3^Fe(CO)_4_ metal center as shown pictorially in Figure 1c.

#### 3.1.1 Chemical Bonding for Singlet and Triplet Irontetracarbonyl

**Figure 2:**
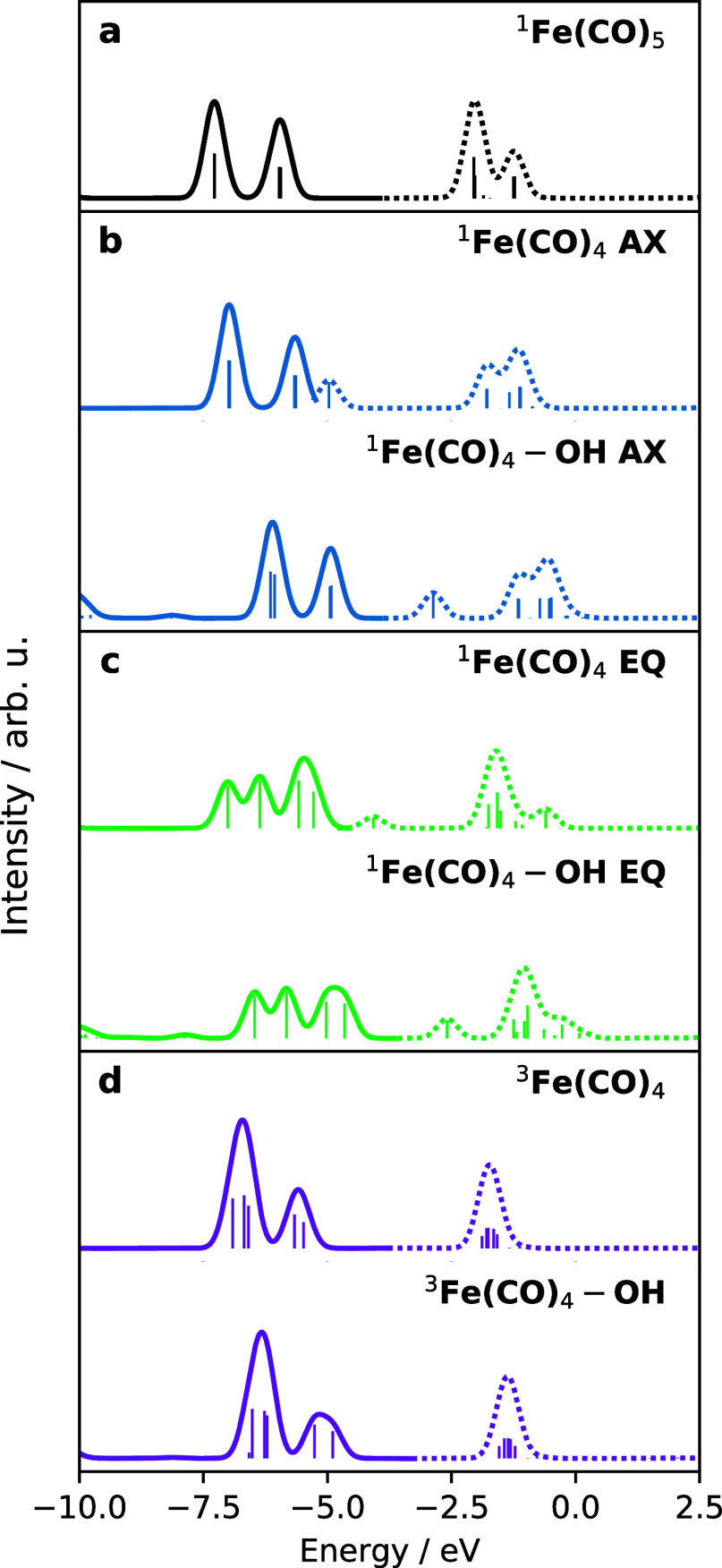
Fe (d) PDOS for different gas-phase complexes where the bold lines denote convoluted occupied orbitals and the dashed lines denote convoluted unoccupied orbitals. With the exception of (a) ^1^Fe(CO)_5_, all panels (b–d) show the optimized structures before and after coordination with an ethanol ligand.

Based on the differences in coordination of Fe(CO)_4_ in different spin states and of ^1^Fe(CO)_5_, we explore the changes in electronic structure by calculations of isolated clusters represented in liquid simulations. To do this, we performed a DOS calculation based on DFT calculations in CP2K. By decomposing the total DOS into projections onto atomic orbital contributions, the total DOS can be analyzed in terms of atom specific PDOS. Figure 2 shows the Fe PDOS, including only the d orbitals. Here, all bold curves denote the occupied orbitals, while all dashed curves denote the unoccupied orbitals.

Figure 2a shows the Fe (d) PDOS of ^1^Fe(CO)_5_ with two distinct peaks in the occupied and unoccupied orbitals. The lowest energy occupied orbitals at −7.0 eV correspond to the degenerate occupied 3d_*xz*_ (e″) and 3d_*yz*_ (e″), followed by the quasi-degenerate  (e′) and 3d_*xy*_ (e′) orbitals at 6.0 eV. The assignment and position of the peaks in the PDOS can be related to previous X-ray photoelectron spectroscopy measurements, with the sequential ordering of  (e′)/3d_*xy*_ (e′) and 3d_*xz*_ (e″)/3d_*yz*_ (e″) occupied orbitals.^40^ The lowest unoccupied orbital at −2.0 eV corresponds to the unoccupied 3d_*z*^2^_ (a_1_^′*^) orbital. The final sets of Fe-containing orbitals correspond to the quasi-degenerate unoccupied π*– (e′*) and π*–3d_*xy*_ (e′*) orbitals at −2.5 eV, followed by the degenerate unoccupied π*–3d_*xz*_ (e″*) and π*–3d_*yz*_ (e″*) orbitals at −1.5 eV. This series of orbital characters is largely preserved for all other complexes shown in Figure 2b–d and allows for the Fe PDOS of ^1^Fe(CO)_5_ to serve as a reference for analyzing the electronic structure of the transient species. The underlying molecular orbitals are depicted in Figures S5–S9.

For all curves in Figure 2b–d, there are two visibly distinct features in the PDOS: (i) there are features associated with the occupied Fe 3d orbitals and (ii) the lowest unoccupied orbital is highly sensitive to coordination. We first consider the top curves in Figure 2b–d that correspond to the optimized singlet ^1^Fe(CO)_4_ and triplet ^3^Fe(CO)_4_, followed by the effect of coordinating an ethanol ligand in the bottom curve in Figure 2b–d. The removal of an axial CO gives the uncoordinated ^1^Fe(CO)_4_ AX, in Figure 2b, which preserves the degeneracies of the occupied band in ^1^Fe(CO)_5_ but pulls down the energy of the lowest unoccupied 3d_*z*^2^_ (a_1_^′*^) orbital. Going from *D*_3h_ symmetry in ^1^Fe(CO)_4_ to ^1^Fe(CO)_4_ AX with C_3v_ symmetry, the a_1_^′*^ orbital transforms to the a_1_^*^ symmetry label, while the occupied e′ and e″ orbitals map onto the symmetry label e. When an equatorial CO is removed, the entire occupied band shifts from a 2-peak feature to a 3-peak feature following the lifting of orbital degeneracies. Relating ironpentacarbonyl to ^1^Fe(CO)_4_ EQ, which possesses *C*_2*v*_ symmetry, the occupied e′ and e″ orbitals are correlated with a_1_, a_2_, b_1_, and b_2_, while the lowest unoccupied orbital, formerly a_1_^′*^, is associated with the symmetry label a_1_^*^. Hoffmann and co-workers^38^ note a peculiarity of the orbital symmetries of ^1^Fe(CO)_4_ EQ, where the LUMO of ^1^Fe(CO)_4_ EQ is composed of a dsp-hybridized orbital possessing strong p-orbital character instead of having a 3d_*z*^2^_ character. Regardless, both orbitals have an a_1_^*^ symmetry. Upon removal of an equatorial CO, the formerly unoccupied 3d_*z*^2^_ in ^1^Fe(CO)_5_ is projected along the *x*-axis, giving an orbital described as π*–3d_*x*^2^_, while the π*– orbital is no longer observed.

The result of these orbital transformations is that ^1^Fe(CO)_5_, ^1^Fe(CO)_4_ AX, and ^1^Fe(CO)_4_ EQ possess a LUMO which has an a_1_ symmetry, and in the latter two complexes, this LUMO is a good σ-accepting orbital capable of bonding with an occupied ligand orbital. Based on this, we find that coordination of ^1^Fe(CO)_4_ by an ethanol ligand dramatically shifts this unoccupied orbital energy as shown in the comparison of spectra for axial coordination in Figure 2c, and likewise for equatorial coordination in Figure 2c. Lastly, in Figure 2d, the number of strong Fe 3d features increased for ^3^Fe(CO)_4_ since the final occupied orbital instead corresponds to the partially filled 3d_*z*^2^_ (a_1_^′*^) orbital arising from the α-spin orbital energies. Coordination of an ethanol ligand to ^3^Fe(CO)_4_ results in a very small change of the PDOS, which is attributed to the weak interaction of the two fragments at a distance as seen in Figure 1a–c. We note that while ^3^Fe(CO)_4_ possesses *C*_2*v*_ (like ^1^Fe(CO)_4_ EQ), the PDOS have a 2-peak feature for the occupied orbitals (like ^1^Fe(CO)_4_ AX). This result is coincidental, where the splitting of the ^1^Fe(CO)_4_ EQ orbital energies in α-spin orbitals results in two peaks in the occupied orbitals, where the formerly unoccupied a_1_^*^ LUMO is filled and shifts into the occupied orbitals at −5.0 eV.

**Figure 3:**
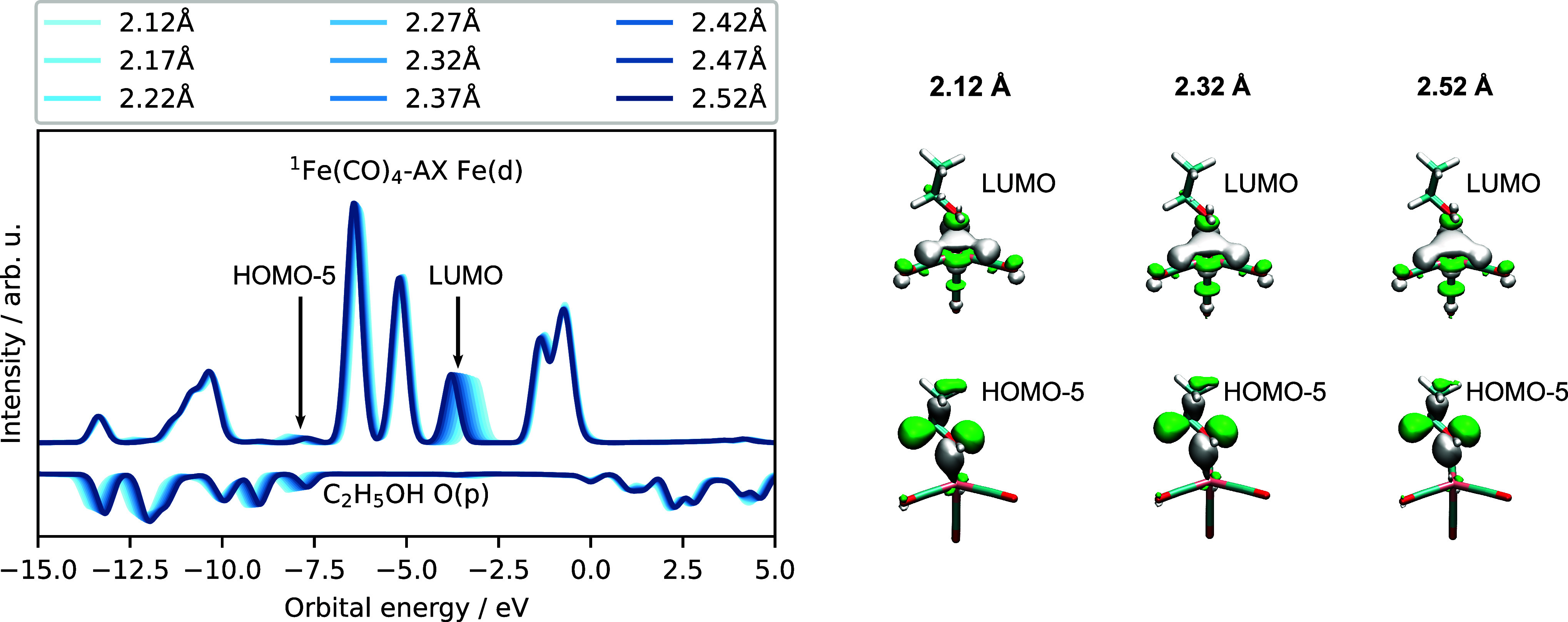
Left: Fe(CO)_4_–OH AX rigid scan with the Fe (d) PDOS and O (p) PDOS for the coordinating hydroxyl oxygen. Right: the changes in the LUMO orbital of predominantly d-character and the HOMO – 5 corresponding to the highest occupied molecular orbital in the ethanol molecule.

To explore the effect of ethanol ligation on the undercoordinated ^1^Fe(CO)_4_, we consider the changes in PDOS along a one-dimensional scan as a function of Fe–O_H_ distance in Figure 3. We do this by elongating the Fe–O_H_ bond starting from the optimized Fe(CO)_4_–OH AX complex from 2.12 to 2.52 Å, representing the distance range from the optimized singlet structure to the point where the spin-contamination in the unrestricted wave function becomes significant. As noted previously, the lowest unoccupied state in the Fe (d) PDOS is particularly sensitive to coordination, resulting from the bonding of the 3d_*z*^2^_ (a_1_^′*^) orbital. In the present scan, we also include the O (p) PDOS corresponding to the ethanol oxygen atom coordinating ^1^Fe(CO)_4_ AX. We note that much like the PDOS for the optimized structures in Figure 1b, the coordination of ethanol produces a shift of the LUMO orbital to higher energies, while all other orbital energies remain unaffected. In addition, we highlight a particularly small but noticeable peak appearing below the lowest four occupied orbitals at roughly −7.5 eV, corresponding to a weak interaction of the Fe d-orbitals with ethanol HOMO – 1. We find that ethanol, unlike ^1^Fe(CO)_4_ AX, experiences a polarization of orbitals that lowers the energies of the frontier molecular orbitals and produces pronounced shifts in the O (p) PDOS, when ethanol experiences the field from the electrophilic iron center. In the right panel of Figure 3, we display the changes in the orbital shape as a function of Fe–O_H_ distance, where it is clear that the small changes of the PDOS are related to small shifts in orbital overlap. At −7.6 eV, the HOMO – 5 orbital of ^1^Fe(CO)_4_–OH AX is a majority combination of the ethanol HOMO with a small degree of overlap with an Fe 3d hybridized orbital. The LUMO orbital of ^1^Fe(CO)_4_–OH AX is in fact originating from the isolated ethanol Fe 3d_*z*^2^_ orbital. As the bond is stretched, we see the large lobe formed by the 3d_*z*^2^_ (a_1_^′*^) orbital of the ^1^Fe(CO)_4_ AX and the reemergence of the oxygen p-orbital, in a minority contribution.

The obvious shifts in PDOS features of the optimized structures prompt structural sampling from the aforementioned AIMD simulations. To do this, we sampled every 100 fs from each AIMD simulation, resulting in 506, 458, and 526 sets of configurations for ^1^Fe(CO)_5_, ^1^Fe(CO)_4_–OH, and ^3^Fe(CO)_4_, respectively. In addition, we partitioned the ^1^Fe(CO)_4_–OH AIMD trajectory into those configurations, which contained only ^1^Fe(CO)_4_–OH AX, where we describe the axial/equatorial comparison in Figure S10.

**Figure 4:**
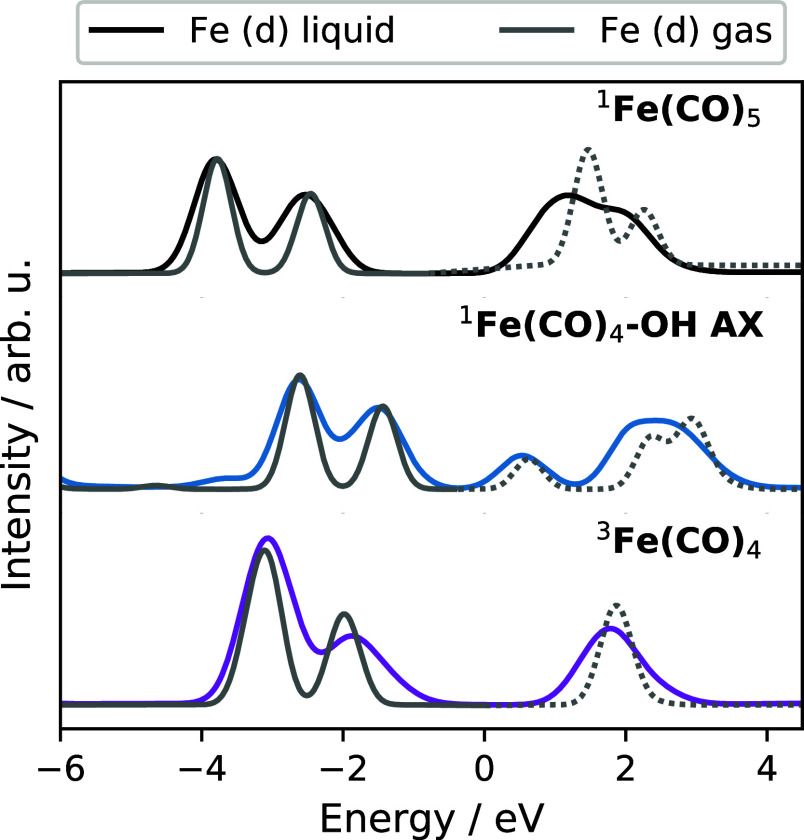
Average Fe (d) PDOS taken every 100 fs from each AIMD simulation. The gas-phase Fe PDOS shown in gray are overlaid to indicate shifts in the peak positions and intensities.

For each sampled configuration, the PDOS were calculated for each atom type in the bulk liquid; however, we only display the Fe (d) PDOS in Figure 4. We overlay the gas-phase PDOS (shown in gray) by shifting the lowest energy feature of the occupied Fe d-orbitals with the corresponding liquid peak. In Figure 4, we find that there is a close agreement with the gas-phase and the bulk liquid PDOS. Also the lowest unoccupied orbitals agree in ^1^Fe(CO)_4_ AX and ^3^Fe(CO)_4_ PDOS. These observations are indicative of a weak interaction with the remaining solvent and validate the CPCM description used in the complementary cluster calculations. We find that the unoccupied orbitals in all curves are polarized by the liquid, which is evidenced by the relative lowering of the unoccupied band energies and intensities.

**Figure 5:**
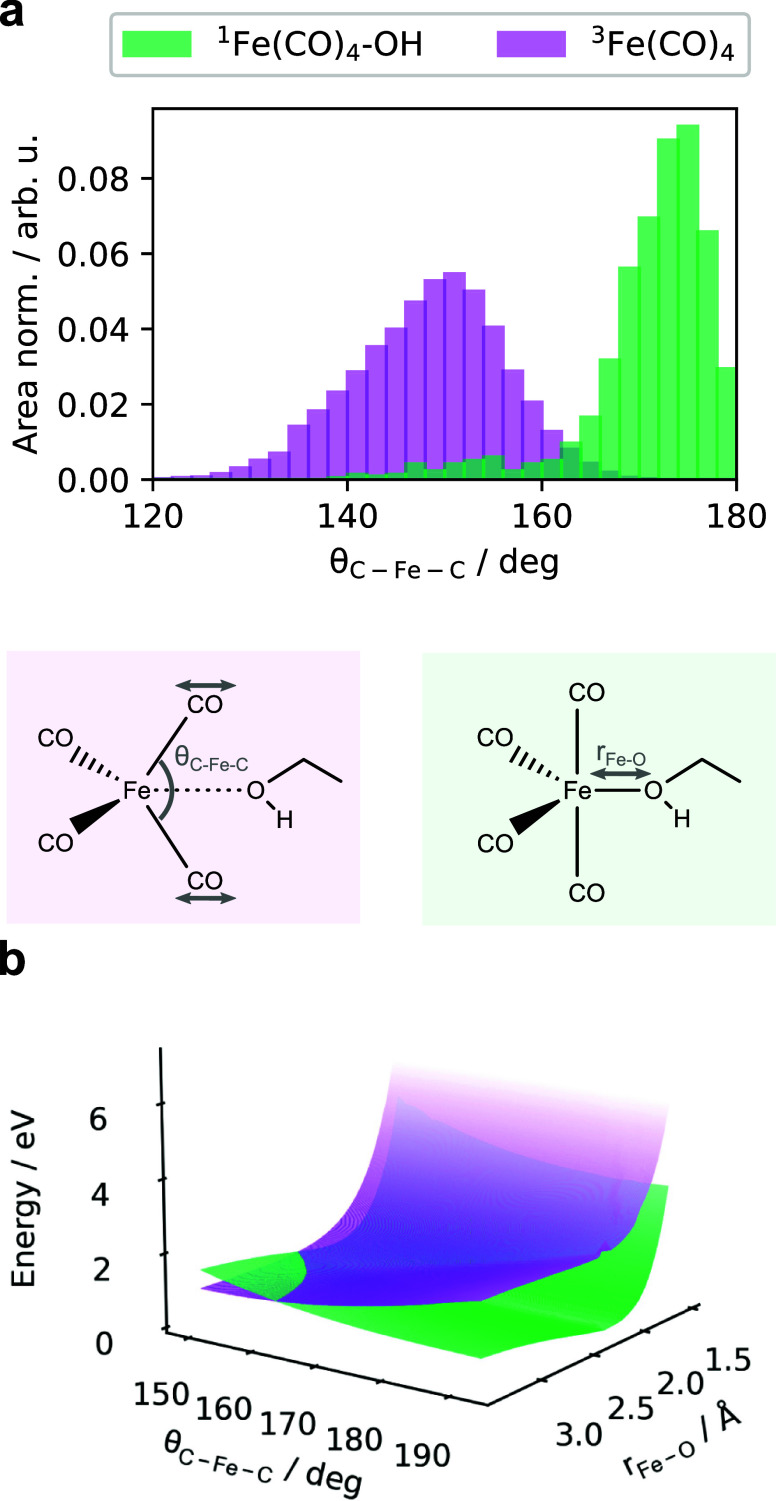
(a) Binned θ_C–Fe–C_ angles for ^1^Fe(CO)_4_–OH EQ and ^3^Fe(CO)_4_ AIMD trajectories. (b) SA(1S+1T)-NEVPT2(10e,10o)/def2-TZVP+CPCM(ethanol) rigid 2-dimensional scan of the axial θ_C–Fe–C_ angle and *r*_Fe–O_ distance of the coordinating ethanol.

Lastly, to relate the singlet and triplet AIMD simulations, we revisit the structural and electronic features of each state. As seen in Figure 1c, there exists a natural connection between ^3^Fe(CO)_4_ and ^1^Fe(CO)_4_–OH EQ by the axial θ_C–Fe–C_ angle and the Fe–O_H_ distance, where the linearity of the axial θ_C–Fe–C_ angle is perturbed by the triplet ground state minimum. From the AIMD simulations, we extract and plot the largest angles from the ^1^Fe(CO)_4_ EQ partitioned set of configurations and from the total ^3^Fe(CO)_4_ trajectory in Figure 5a. Here, each distribution is area-normalized to be able to compare the peak intensities of the distributions. This is due to the simulation time lengths being different in the ^1^Fe(CO)_4_ EQ and ^3^Fe(CO)_4_ trajectories and, hence, the different numbers of sampled configurations. In agreement with the optimized structures, the axial θ_C–Fe–C_ angle in ^1^Fe(CO)_4_–OH EQ is predominantly linear (calcd: 179° from structure library) in contrast to ^3^Fe(CO)_4_, which has a predominantly bent axial θ_C–Fe–C_ angle (calcd: 150.6° from structure library). We note from Figure 1a that there is a distinct shift in Fe–O_H_ distance, while Figure 1b indicates that the singlet state is, on average, lower in energy than the triplet state. In Figure 5b, we show the rigid 2D scan along a varying θ_C–Fe–C_ angle and the Fe–O_H_ distance. In the top panel of Figure 5b, the definitions of the two coordinates are shown schematically on representative structures of the two minima, a triplet in purple and a singlet in green. In the potential energy surfaces, we see that a contribution to the repulsive nature of the triplet state is related to a repulsive potential energy surface at short Fe–O_H_ distances. Furthermore, the surfaces indicate that based on the scanned degrees-of-freedom, the triple surface is generally higher in energy with a crossing seam at longer Fe–O_H_ distance and bent axial θ_C–Fe–C_ angles.

### 3.2 Solvent Coordination to Singlet Irontetracarbonyl

**Figure 6:**
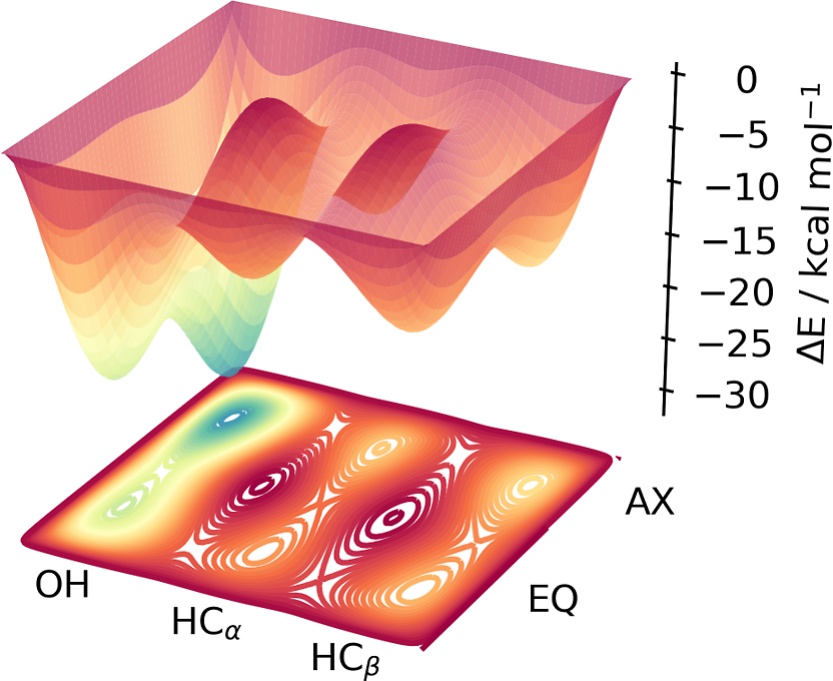
Schematic of minima in the singlet ^1^Fe(CO)_4_ based on energies calculated at the DLPNO-CCSD(T)/def2-TZVP level of theory based on structures optimized at the TPSSh/def2-TZVP + CPCM(ethanol) level of theory. The energies are taken relative to the sum of the ^1^Fe(CO)_4_ EQ and ethanol optimized structures. These energies are summarized in Table 1.

We have shown in Section 3.1, the global minima of ^1^Fe(CO)_4_–OH in comparison to the ^1^Fe(CO)_5_ and ^3^Fe(CO)_4_ AIMD simulations to highlight the differences between the reactant and final photoproducts in each spin state. Complementary to the simulations results, there is an extensive literature of static calculations investigating the various bonding types within the reactive singlet states of under-coordinated 3d metal carbonyl complexes. Figure 6 provides a schematic for the various minima and transition state energies in the last column of Table 1.

**Table 1:** Relative Energies of Minima and Transition States, Denoted with ‡, of the Coordinations of an Ethanol Ligand with ^1^Fe(CO)_4_ Based on Structures Optimized at the TPSSh/def2-TZVP + CPCM(ethanol) Level of Theory^a^

	TPSSh (Gaussian)	BLYP (CP2K)	NEVPT2 (ORCA)	DLPNO-CCSD(T) (ORCA)
^1^Fe(CO)_4_–AX	0.00	0.00	0.00	0.00
^3^Fe(CO)_4_	–7.33	–7.64	–1.81	3.66
^1^Fe(CO)_4_–OH AX	–23.49	–27.83	–30.39	–27.43
^3^Fe(CO)_4_···OH^b^	–10.16	–9.44	–2.92	3.62
^1^Fe(CO)_4_–OH EQ	–19.27	–24.91	–29.65	–19.20
^1^Fe(CO)_4_–HC_α_ AX	–8.75	–15.55	–13.85	–10.53
^1^Fe(CO)_4_–HC_α_ EQ	–9.29	–15.89	–16.45	–8.10
^1^Fe(CO)_4_–HC_β_ AX	–7.68	–12.18	–13.14	–10.61
^1^Fe(CO)_4_–HC_β_ EQ	–8.90	–14.14	–17.51	–8.37
[^1^Fe(CO)_4_–OH]^‡^ AX/EQ	–18.81	–23.35	–26.35	–17.48
[^1^Fe(CO)_4_–HC_α_]^‡^ AX/EQ	–6.71	–14.31	–12.43	–5.55
[^1^Fe(CO)_4_–HC_β_]^‡^ AX/EQ	–5.90	–10.54	–12.31	–5.40
[^1^Fe(CO)_4_–HC_α_/OH]^‡^ AX	–1.70	–7.64	–6.50	–4.42
[^1^Fe(CO)_4_–HC_α_/OH]^‡^ EQ	–4.94	–12.22	–12.19	–3.33
[^1^Fe(CO)_4_–HC_β_/OH]^‡^ AX	–3.17	–10.03	–7.28	–3.64
[^1^Fe(CO)_4_–HC_β_/OH]^‡^ EQ	–6.52	–14.61	–13.37	–4.54
[^1^Fe(CO)_4_–HC_α_/HC_β_]^‡^ AX^b^	1.17	–7.24	–3.71	–1.94
[^1^Fe(CO)_4_–HC_α_/HC_β_]^‡^ EQ	–5.13	–11.08	–8.72	–1.62

a The uncoordinated ^1^Fe(CO)_4_ AX, ^1^Fe(CO)_4_ EQ, and ^3^Fe(CO)_4_ all include the energy based an optimized ethanol molecule. All structures are taken relative to the ^1^Fe(CO)_4_ EQ + ethanol energy. The BLYP energies calculated using CP2K used a cell size of 30 Å to prevent interactions with all other periodic images without CPCM. The NEVPT2 energies are based on a CAS(10e, 10o)/def2-TZVP + CPCM(ethanol) reference calculation in ORCA. The coupled cluster energies are based on the DLPNO-CCSD(T)/ZORA-def2-TZVP + CPCM(ethanol) level of theory using ORCA. All values are reported in kcal mol^–1^. The double lines distinguish the geometries considered in Section 3.1 and those within the singlet coordination in Section 3.2.

b See the Supporting Information for details about these structures.

Here, the DLPNO-CCSD(T)/def2-TZVP + CPCM(ethanol) energies were calculated for all of the singlet structures within the structure library. For comparison, also TPSSh, BLYP, and NEVPT2 energies are given but not discussed. Furthermore, a comparison of the BLYP and B3LYP functionals is presented in Table S1. To verify the small multiconfigurational character of each complex, the configuration state function (CSF) weights obtained from the CASSCF optimization are presented in Table S2. We find that all of the complexes are described by a single electron configuration (i.e., the closed shell singlet or open shell triplet), with a leading CSF weight (*c*_0_^2^) exceeding 0.8. All energies are taken with reference to the sum of the ^1^Fe(CO)_4_ AX and ethanol energies. While the gas-phase global minimum of ^1^Fe(CO)_4_ has a ligand vacancy in an equatorial position of ^1^Fe(CO)_5_, the ethanol ligated complexes all have a deeper minimum in an axial configuration. The difference in ^1^Fe(CO)_4_ AX and ^1^Fe(CO)_4_ EQ in Table 1 is on the order of 1.0 kcal mol^–1^. The schematic in Figure 6 represents possible connectivity of the transition states and minima, where the box boundaries are set to 0.0 kcal mol^–1^ (the ^1^Fe(CO)_4_ AX energy in Table 1). Furthermore, no diagonal transition states are included, i.e., ^1^Fe(CO)_4_–OH AX to ^1^Fe(CO)_4_–HC_α_ AX represents a barrier but ^1^Fe(CO)_4_–OH AX to ^1^Fe(CO)_4_–HC_α_ EQ does not. These diagonal crossings are set to 0.0 kcal mol^–1^, which are shown as the two peaks in the center of the schematic.

As previously discussed, the ^1^Fe(CO)_4_–OH AX complex is the lowest energy complex with a Δ*E*_int_ = −27.43 kcal mol^–1^, while the corresponding ^1^Fe(CO)_4_–OH EQ complex is roughly 8.0 kcal mol^–1^ higher in energy with a Δ*E*_int_ = −19.20 kcal mol^–1^. The corresponding optimized transition state energy between the two minima is at Δ*E*^⧧^ = −17.48 kcal mol^–1^, roughly 2.0 kcal mol^–1^ above ^1^Fe(CO)_4_–OH EQ and roughly 10.0 kcal mol^–1^ above ^1^Fe(CO)_4_–OH AX. Within the bulk liquid at 300 K, the fluctuations in the potential energy in Figure 1b are on the order of 50.0 kcal mol^–1^, providing a basis for the sampling of both ^1^Fe(CO)_4_–OH AX and ^1^Fe(CO)_4_–OH EQ minima. So far, the discussion of the results has centered on the equilibrium between ^1^Fe(CO)_4_–OH AX and ^1^Fe(CO)_4_–OH EQ in the AIMD simulations. However, the coordination with the alkyl chain of ethanol is possible.^5^ We see in Figure 6 and Table 1 that the coordination with the α-carbon and β-carbon gives four minima elevated at least 15 kcal mol^–1^ above the ^1^Fe(CO)_4_–OH AX minimum. C–H coordination of ethanol is compared to that of ^1^Fe(CO)_4_; coordination at the β-carbon is slightly less strong than at the α-carbon. The lowest energy –CH coordination is the ^1^Fe(CO)_4_–HC_β_ AX complex with Δ*E*_int_ = −10.61 kcal mol^–1^, followed closely by the ^1^Fe(CO)_4_–HC_α_ AX complex with Δ*E*_int_ = −10.53 kcal mol^–1^. We therefore expect overlapping distributions of the relative energies in AIMD simulations of these two types of bonding.

**Figure 7:**
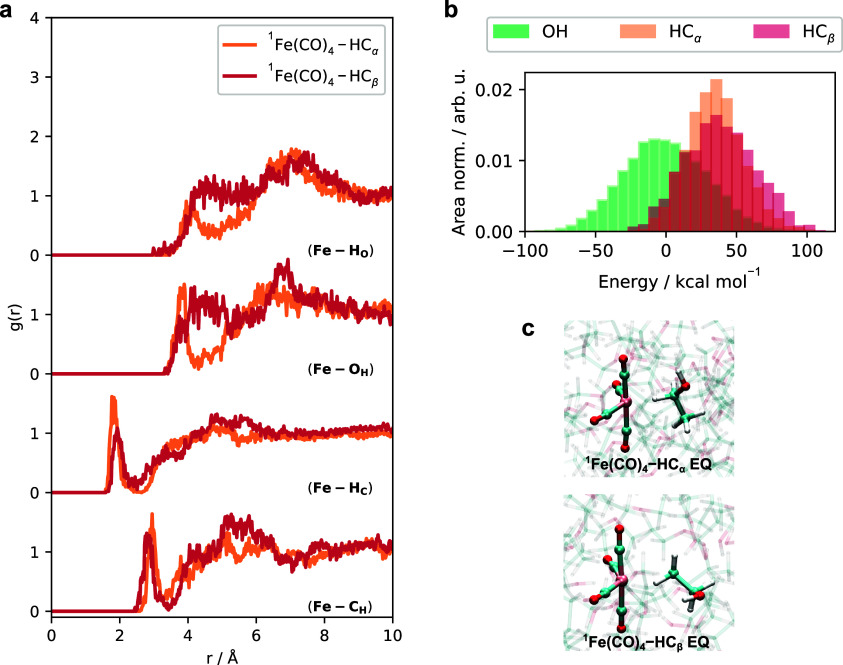
(a) Radial distribution functions *g*(*r*) sampled from the ^1^Fe(CO)_4_–HC_α_ and ^1^Fe(CO)_4_–HC_β_ simulations. The coordination around the iron complexes are seen in *g*(*r*) for Fe–H_O_, Fe–O_H_, Fe–H_C_, and Fe–C_H_. The distances from ^1^Fe(CO)_4_–HC_α_ are colored orange and from ^1^Fe(CO)_4_–HC_β_ are colored red. For clarity, the individual *g*(*r*) classes are plotted separately in Figure S11 alongside the ^1^Fe(CO)_4_–OH *g*(*r*) reproduced from Figure 1. (b) Distribution of the potential energies from the ^1^Fe(CO)_4_–OH (reproduced from Figure 1), ^1^Fe(CO)_4_–HC_α_, and ^1^Fe(CO)_4_–HC_β_ simulations, following the same color scheme. All energy distributions are area-normalized to account for the differences in simulation length. (c) Representative snapshots from the AIMD simulations for the two distinct minima observed between the Fe–HC coordinated simulations.

To assess the stability of these transient ^1^Fe(CO)_4_–HC_α_ and ^1^Fe(CO)_4_–HC_β_ minima, we re-equilibrated the ^1^Fe(CO)_4_–OH AIMD trajectory to allow for the formation of a Fe–C_H_ bond by means of a restraint of the Fe–C_H_ bond distances. We did so because initial attempts to fix the Fe–C_H_ bond distance and release the AIMD trajectory failed to create even a metastable configuration and rapidly converted back to the ^1^Fe(CO)_4_–OH minima. In the results presented in Figure 7, we equilibrated both –CH minima with a restraint, followed by a release of the restraint and a subsequent evolution of the trajectory for an additional 15 ps. In Figure 7a, we show the *g*(*r*) for the same pairs of atoms as in Figure 1a but for the ^1^Fe(CO)_4_–HC_α_ (orange) and ^1^Fe(CO)_4_–HC_β_ (red) AIMD simulations. The –CH coordinated trajectories show narrow peaks centered around 2.1 Å in the Fe–H_C_*g*(*r*) and 2.7 Å for the Fe–C_H_*g*(*r*), indicating the formation of the Fe–C_H_ bond. The bonding via the –CH group occurs principally via the hydrogen to the carbon via the σ_CH_ orbital;^41,42^ hence, the Fe–H_C_ distances are on average shorter.

Additionally, we find that while the Fe–H_C_*g*(*r*) have peaks essentially centered around the same distance at 2.7 Å, the presence of the –OH group near the α-carbon causes a lengthening of the C–H bond and an increase in the θ_Fe–H–C_ to give a longer Fe–C_H_ bond. This interpretation of the results is corroborated with the structure library, which shows Fe–C_H_ bond lengths of 2.79 and 2.59 Å, θ_Fe–H–C_ angles of 142.6 and 119.2° for ^1^Fe(CO)_4_–HC_α_ EQ and ^1^Fe(CO)_4_–HC_β_ EQ, respectively.

In the –CH coordinated trajectories, we find no clear coordination with the hydroxyl group; this is shown by the Fe–H_O_ and Fe–O_H_*g*(*r*) ,which have a first coordination shell beginning at roughly 3.4 and 3.7 Å for the Fe–H_O_ and Fe–O_H_ coordination, respectively. Instead there is an ordering of the first solvation shell with respect to the identity of the coordinating alkyl –CH bond type. For those configurations, coordinating via the α-carbon, the Fe–H_O_ and Fe–O_H_*g*(*r*) show sharp peaks centered around 3.8 Å, corresponding to the distance between the iron and the oxygen of the coordinating ethanol ligand. Additionally, the Fe–H_O_ and Fe–O_H_*g*(*r*) values are more peaked for the configurations from the ^1^Fe(CO)_4_–HC_α_ trajectory because the distance between the iron and oxygen is connected via the α-carbon and is only determined by the θ_Fe–C–O_ angle. By contrast, the distance between the iron and oxygen in the ^1^Fe(CO)_4_–HC_β_ trajectory is connected via the β-carbon and α-carbon by the θ_Fe–C–C–O_ dihedral angle, which at a small angle gives a short Fe–O_H_ distance or at a large angle gives a large Fe–O_H_ distance. This type of bonding has been presented in the structure library, where the small θ_Fe–C–C–O_ dihedral angle connects the ^1^Fe(CO)_4_–HC_β_ structure to the ^1^Fe(CO)_4_–OH structure via a transition state.

In Figure 1b, the distributions of potential energies for ^1^Fe(CO)_4_–OH, ^1^Fe(CO)_4_–HC_α_, and ^1^Fe(CO)_4_–HC_β_ are displayed, with the energies taken with respect to the average energy of the ^1^Fe(CO)_4_–OH distribution. We find that the distributions of ^1^Fe(CO)_4_–HC_α_ and ^1^Fe(CO)_4_–HC_β_ energies are shifted approximately 50 kcal mol^–1^ from the ^1^Fe(CO)_4_–OH distribution. It should be noted that the –CH coordinated distributions appear to overlap with the distribution of the ^3^Fe(CO)_4_ energies in Figure 1b. We note, however, that the trajectories are not sampling the same coordination space, as evidenced by the differences in the radial distribution functions in Figures 1a and7a. As previously stated, we expected, based on the structure library and energies from Figure 6 and Table 1, that the energetic distinction between types of –CH coordination would be limited and we find that the resulting potential energy distributions are strongly overlapping. Finally, while we find an explicit sampling of both axial and equatorial coordinations of ^1^Fe(CO)_4_–OH in the AIMD simulations, we only sample the equatorial minima with the present set of trajectories as shown in Figure 7c.

**Figure 8:**
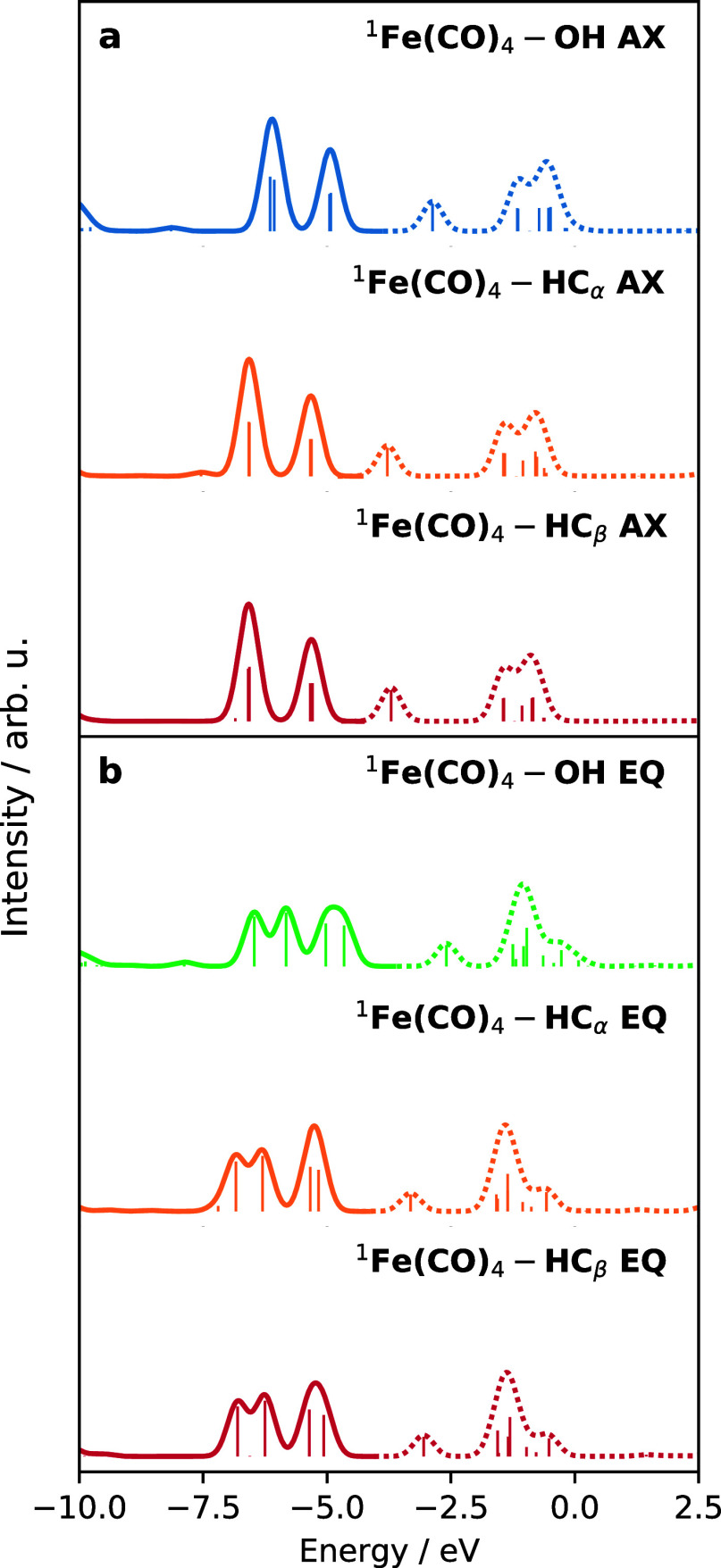
Fe (d) PDOS for different gas-phase complexes where the bold lines denote convoluted occupied orbitals and the dashed lines denote convoluted unoccupied orbitals. (a) Three distinct axially coordinated minima ^1^Fe(CO)_4_–OH AX (blue), ^1^Fe(CO)_4_–HC_α_ (orange), and ^1^Fe(CO)_4_–HC_β_ (red). (b) Three distinct equatorial coordinated minima ^1^Fe(CO)_4_–OH AX (green), ^1^Fe(CO)_4_–HC_α_ (orange), and ^1^Fe(CO)_4_–HC_β_ (red). Following the same convention previously stated, the bold lines denote convoluted occupied orbitals and the dashed lines denote convoluted unoccupied orbitals.

Based on the different coordinations of ^1^Fe(CO)_4_–OH, ^1^Fe(CO)_4_–HC_α_, and ^1^Fe(CO)_4_–HC_β_ in the structure library and those structures observed in the AIMD simulations, we perform the same PDOS analysis done in Figure 2 for the minima in the singlet state and display the results in Figure 8. Given the clear distinction between the PDOS of ^1^Fe(CO)_4_ AX and ^1^Fe(CO)_4_ EQ in Figure 2c,d, we divide the PDOS based on the structure library into the axial and equatorial coordinations in Figure 8a,b, respectively. In addition, we reproduce the PDOS from Figure 2c,d to complete the set of calculations on the minima in the structure library and to draw a contrast between different types of coordination. We find that the 2-peak (axial) and 3-peak (equatorial) feature of the occupied orbitals is preserved across all structures, indicating that the occupied band is not significantly influenced by the coordination of the ethanol, rather the orbital symmetries of the parent complex ^1^Fe(CO)_4_ in the axial or equatorial configuration dictates the shifts in peaks. Since the occupied orbitals that give rise to the sticks in the PDOS are predominantly of Fe (d) character, the coordination of an ethanol is not expected to change the occupied band significantly. Instead, we find that the various singlet minima produce large shifts between the coordination via the –CH or –OH group. Here, the –CH coordinating PDOS produces a small shift of the lowest unoccupied state when compared to the corresponding parent ^1^Fe(CO)_4_ complex versus the –OH coordination. The relative difference in peak position for the lowest unoccupied state between the –CH and –OH configurations is roughly 1.5 eV. The shift of the LUMO orbital of ^1^Fe(CO)_4_–HC_α_ and ^1^Fe(CO)_4_–HC_β_ is small from the corresponding parent ^1^Fe(CO)_4_ complex, indicating that the orbital interaction is weaker than the corresponding ^1^Fe(CO)_4_–OH coordination. Generally, –CH coordination is weak relative to –OH coordination^13,43^ and from a total energy picture, the –CH coordinated complexes are less stable than the corresponding –OH coordinated complexes. Lastly, the distinction between axial and equatorial coordination is preserved across all kinds of –CH or –OH coordination, where the unoccupied orbitals are not sensitive to the choice of ethanol coordination. Again, this is related to the character of the underlying orbitals, which are of a mixed π_CO_^*^–d character (see Section 3.1).

### 3.3 Solvent–Solute Interactions

In Section 3.1, we showed that there was a clear distinction between the coordination of ethanol in the singlet and triplet states, where the singlet state forms an explicit covalent bond between ^1^Fe(CO)_4_ and an ethanol solvent molecule, while the triplet state produces an unreactive ^3^Fe(CO)_4_ that preferentially interacts with a single ethanol solvent molecule. We also found in Section 3.2 that ^1^Fe(CO)_4_ forms explicit bonds via the –OH or –CH coordination, where the latter is a particularly unique type of covalent bonding within organometallic chemistry.^41,42^ Here, we use DLPNO-CCSD(T) calculations on the ^1^Fe(CO)_4_–OH AX, ^1^Fe(CO)_4_–HC_α_ AX, and ^3^Fe(CO)_4_ structures in the solvent library to quantify the electronic origins for the bonding or lack of bonding in each coordination and state. To do so, we follow the LED procedure outlined by Bistoni et al.,^39^ which we define in Section 2.2. The results of the LED analysis performed on energies calculated at the DLPNO-CCSD(T)/def2-TZVP + CPCM(ethanol) level of theory are summarized in Table 2.

**Table 2:** Results of the LED Analysis Calculated at the DLPNO-CCSD(T)/def2-TZVP Level of Theory^a^

	Δ*E*_int_ (CPCM)	Δ*E*_int_	Δ*E*_geo-prep_	Δ*E*_el-prep_^HF^	*E*_elstat_^HF^	*E*_exch_^HF^	Δ*E*_non-disp_^C-CCSD^	Δ*E*_disp_^C-CCSD^	Δ*E*_int_^C-(T)^
^1^Fe(CO)_4_–OH	–27.43	–26.84	1.73	190.17	–177.25	–26.87	–2.83	–9.23	–2.55
^1^Fe(CO)_4_–HC_α_	–10.53	–11.08	4.07	153.20	–126.31	–24.68	–6.39	–8.47	–2.49
^3^Fe(CO)_4_···OH^b^	3.62	1.50	3.02	2.87	–1.83	–0.86	–0.41	–1.08	–0.19
^1^Fe(CO)_4_···OH^b^		1.71	3.71	13.19	–11.55	–2.50	0.30	–1.59	0.15

a All calculations were performed on structures optimized at the TPSSh/def2-TZVP + CPCM(ethanol) level of theory. All energies are displayed in units of kcal mol^–1^. ^1^Fe(CO)_4_···OH denotes the calculation of ^3^Fe(CO)_4_···OH in the singlet state. The interaction energies calculated with CPCM(ethanol) from Table 1 are included to highlight the effect of implicit solvation.

b See the Supporting Information for details about these structures.

For the first two species (^1^Fe(CO)_4_–OH and ^1^Fe(CO)_4_–HC_α_), we find that the electrostatic components (Δ*E*_el-prep_HF and *E*_elstat_^HF^) of the interaction energy are large but largely cancel each other out. For ^1^Fe(CO)_4_–OH, the Δ*E*_el-prep_^HF^ energy that is calculated from the HF energies is the largest for all complexes studied. By taking the sum of Δ*E*_geo-prep_, Δ*E*_el-prep_^HF^, *E*_elstat_^HF^, and *E*_exch_^HF^, we find that the net interaction energy is negative for ^1^Fe(CO)_4_–OH (−12.23 kcal mol^–1^) and net positive for ^1^Fe(CO)_4_–HC_α_ (6.27 kcal mol^–1^). This follows from the fact that the Fe–O_H_ bond is more covalent than the Fe–C_H_ bond, which forms a weak bond between an iron MC orbital and a σ(C–H) molecular orbital on the ethanol. For ^1^Fe(CO)_4_–OH, the nondispersive energy correction is small but attractive, while the dispersive component is larger and also attractive. For ^1^Fe(CO)_4_–HC_α_, the results of the summed terms above amounting to a repulsive energy of 6.27 kcal mol^–1^ are nearly canceled out by the attractive nondispersive dynamical correlation corrections (Δ*E*_non-disp_^C-CCSD^) to the HF energies. The remaining dispersive energy therefore constitutes a much larger contribution to the total interaction energy for ^1^Fe(CO)_4_–HC_α_ than that for ^1^Fe(CO)_4_–OH.

We contrast the singlet species which form tightly bound complexes with the looser bonding for the triplet ^3^Fe(CO)_4_ complex. To do so, we perform LED analysis on the optimized ^3^Fe(CO)_4_···OH structure in the triplet and singlet states. We note that for both states, the interaction energy at the DLPNO-CCSD(T)/def2-TZVP + CPCM(ethanol) level of theory is small and positive, indicating that the interaction is weakly repulsive. Since the triplet state is unreactive but weakly coordinating an ethanol solvent molecule, the preparation energy terms (Δ*E*_geo-prep_ and Δ*E*_el-prep_^C-CCSD^) and additional terms arising from the HF (*E*_elstat_^HF^, *E*_exch_^HF^) are small. By taking the sum of Δ*E*_geo-prep_, Δ*E*_el-prep_^HF^, *E*_elstat_^HF^, and *E*_exch_^HF^, we find that the net energy is positive for ^3^Fe(CO)_4_···OH (3.20 kcal mol^–1^) and ^1^Fe(CO)_4_···OH (2.86 kcal mol^–1^), indicating a repulsive interaction. This means that the attractive interaction of the two fragments is dominated by the dynamical correlation corrections coming from the CCSD(T) terms. The largest of these terms in the triplet state is the attractive dispersion energy term followed by the smaller attractive nondispersion energy term. In the singlet state, only the dispersion energy term is attractive, indicating the importance of dispersion interactions in the association of an ethanol solvent molecule to the unreactive triplet ^3^Fe(CO)_4_.

### 3.4 Ligand Exchange

**Figure 9:**
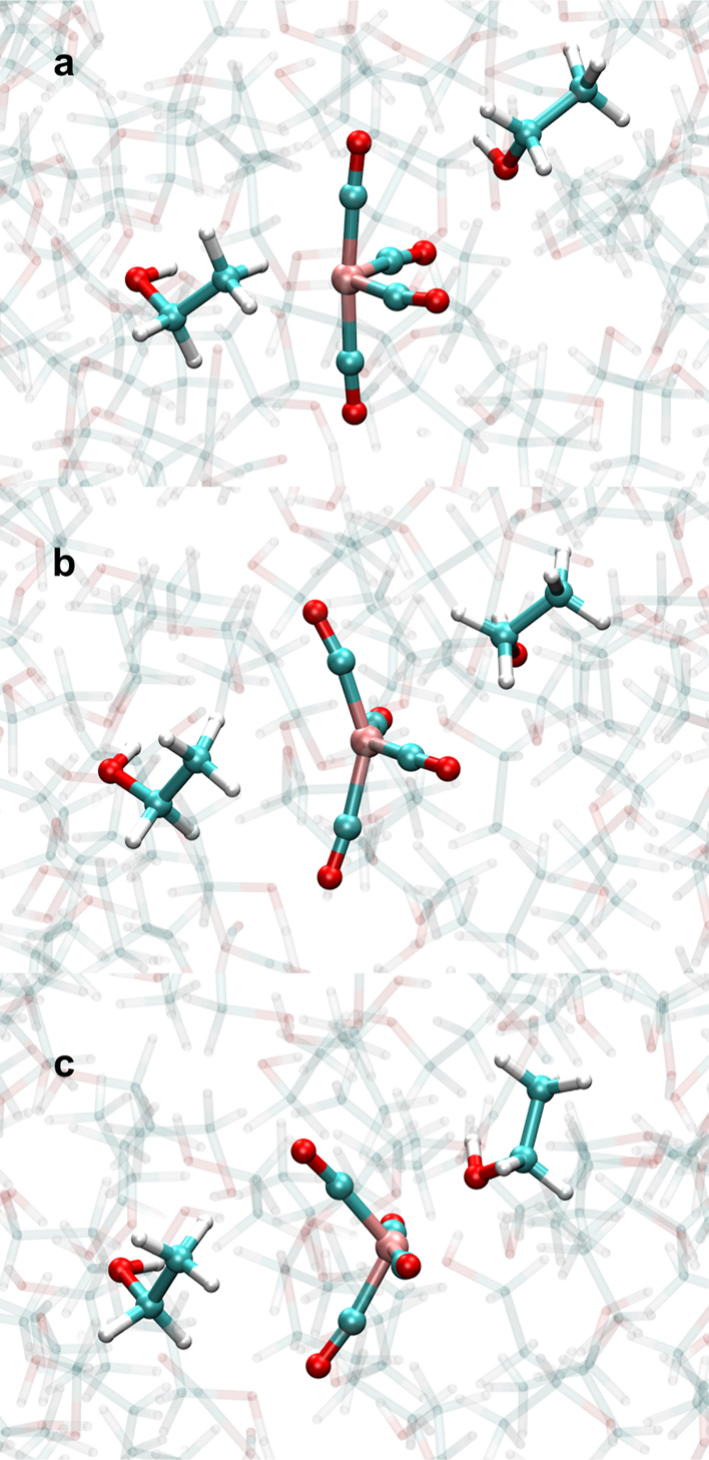
Snapshots of the observed intermolecular ligand exchange mechanism of ^1^Fe(CO)_4_–HC_β_ to ^1^Fe(CO)_4_–OH. (a) ^1^Fe(CO)_4_–HC_β_ at 19.14 ps. (b) Representative snapshot of the bimolecular transition state geometry at 19.40 ps. (c) ^1^Fe(CO)_4_–OH at 19.57 ps.

In Section 3.2, the relative energies of the minima and transition states of ^1^Fe(CO)_4_–EtOH are shown and discussed in terms of the intramolecular conversion between the different configurations. The intramolecular rearrangement follows a “chain-walking” mechanism, whereby a single ligand with multiple coordination sites is capable of reacting via a higher-energy configuration followed by an energetically favorable conversion to a lower-energy configuration. The AIMD simulations have a limited sampling of ligand rearrangements (i.e., pseudorotation), and we find no instance of intramolecular rearrangement within the short simulation times. By contrast, the only instance of ligand rearrangement occurs via an intermolecular mechanism of solvent exchange at the end of the ^1^Fe(CO)_4_–HC_β_ AIMD simulation. In Figure 9, we display representative snapshots of the solvent exchange mechanism that occurs via a backside attack of the ^1^Fe(CO)_4_ backbone.

In Table 1, we showed for irontetracarbonyl interacting with a single solvent molecule that the ^1^Fe(CO)_4_–OH species were always energetically more favorable than the corresponding ^1^Fe(CO)_4_–CH coordinated complexes. Transition states of exchange of coordination within the complex were also determined. However, the AIMD simulations showed an event that inspired us to extend the exchange process to involve two ethanol molecules. At the end of the AIMD simulation of ^1^Fe(CO)_4_–HC_β_, we observed that a backside attack of ^1^Fe(CO)_4_ by a second ethanol solvent molecule via the hydroxyl group occurs within roughly 300 fs and thereby terminated the ^1^Fe(CO)_4_–HC_β_ AIMD simulation by yielding the energetically favored ^1^Fe(CO)_4_–OH species (see Figures 1b and 7b). In order to quantify the transition state energy of a two solvent molecule exchange from Fe–HC_β_ to Fe–OH coordination, we performed a nudged elastic band (NEB)^44^ calculation of the reaction coordinate with 15 beads in order to search for the quasi-transition state structure. The optimized ^1^Fe(CO)_4_–HC_β_ + EtOH and ^1^Fe(CO)_4_–OH + EtOH complexes and transition pathway, found with NEB, were calculated in relation to the species in Table 1 and are summarized in Table 3.

**Table 3:** Relative Energies of Minima and Transition State, Denoted with ‡, Involved in the Intermolecular Solvent Exchange Mechanism Calculated at the DLPNO-CCSD(T)/def2-TZVP + CPCM(ethanol) Level of Theory^a^

	Δ*E*
^1^Fe(CO)_4_–HC_β_ + EtOH	–9.05
[C_β_H–^1^Fe(CO)_4_–OH]^‡^	1.63
^1^Fe(CO)_4_–OH + EtOH	–19.55

a All calculations were performed on structures optimized at the TPSSh/def2-TZVP level of theory without CPCM. All energies are displayed in units of kcal mol^–1^.

We find that the presence of an additional nonbonding ethanol molecule has only a small effect on the relative energies of each complex, with values of −9.05 kcal mol^–1^ (−8.37 kcal mol^–1^ in Table 1) and −19.20 kcal mol^–1^ (−19.55 kcal mol^–1^ in Table 1) for ^1^Fe(CO)_4_–HC_β_ + EtOH and ^1^Fe(CO)_4_–OH + EtOH, respectively. In contrast, the NEB optimized transition state is predicted to be much higher, where the relative energy of the transition state is 1.63 kcal mol^–1^ relative to −4.54 kcal mol^–1^ for the same type of reaction ([^1^Fe(CO)_4_–HC_β_/OH]^‡^ EQ in Table 1). This gives competing barrier heights of 3.83 kcal mol^–1^ for the intramolecular rearrangement and 10.68 kcal mol^–1^ for the intermolecular rearrangement reaction under the assumption that the surrounding solvent is not changing the balance. We note that our limited sampling based on short simulation times prohibits the possibility of providing a statistically relevant sampling of an intra- vs intermolecular rearrangement. We find that while the implicitly solvated optimizations predict an energetically repulsive transition state, higher in energy than the free ^1^Fe(CO)_4_, the solvent interactions of the bulk liquid show a dynamically preferred pathway for decay.

## 4 Conclusions

Through AIMD simulations and quantum chemistry calculations, we have seen how the transient species from photoexcitation of Fe(CO)_5_ in ethanol solution sample various local minima and have proposed the possible mechanisms for the interconversion between the minima. In particular, the essential differences between the parent complex, Fe(CO)_5_ and the main photo products in the singlet (^1^Fe(CO)_4_–OH AX) and triplet (^3^Fe(CO)_4_···OH) states have been detailed, where we find that Fe(CO)_5_ does not coordinate any solvent molecules, while ^1^Fe(CO)_4_–OH AX tightly binds an ethanol molecule via the –OH group and ^3^Fe(CO)_4_···OH weakly coordinates a solvent molecule via electrostatic interactions. The structural differences connecting the singlet and triplet ground states are indicative of a potential relaxation pathway of the triplet state. It has been noted previously^7,45^ and shown here in Table 1 that the electronic ground state of isolated Fe(CO)_4_ is the triplet state, formed following the photodissociation of Fe(CO)_5_. The present AIMD simulations provide a structural argument for the ISC that results in a bound Fe(CO)_4_–OH complex in the singlet state. In addition, through AIMD simulation, we consider different bonding arrangements of ^1^Fe(CO)_4_ and find higher energy local minima of hydroxyl-coordinated ^1^Fe(CO)_4_ via both carbon coordination sites in ethanol.

In order to relate the changes in configuration to chemical bonding, we extended the study to a PDOS analysis of implicitly solvated optimized structures and a sampling of the configurations obtained from the AIMD simulations. Our results showed that the observed trends of axial and equatorial coordination and hydroxyl and alkyl coordination in the gas-phase PDOS were preserved in the sampled liquid trends and suggest that these trends would be experimentally verifiable.

Lastly, we have proposed two types of isomerization mechanisms in the singlet manifold involving either an intramolecular rearrangement (obtained from an accurate implicitly solvated structure search) or an intermolecular exchange mechanism (obtained from the AIMD simulation). These results indicate the possibility of a dynamically preferred pathway in the liquid (exchange mechanism) over the conventionally proposed intramolecular “chain-walking” mechanism.

## Data Availability

The data sets generated and analyzed during the current study are available from the corresponding author on reasonable request and can be accessed on Zenodo. An initial draft of the manuscript was deposited on the ChemRxiv preprint repository.^46^

## Supporting Information Available

CASSCF active space description of Fe(CO)_5_, individual RDF plots of ^1^Fe(CO)_5_, ^3^Fe(CO)_4_ and ^1^Fe(CO)_4_–OH, angle exchange mechanism, ^3^Fe(CO)_4_ solvent interactions, plot of KS-DFT molecular orbitals for the ^1^Fe(CO)_5_, ^1^Fe(CO)_4_ AX, ^1^Fe(CO)_4_ EQ, and ^3^Fe(CO)_4_ structures, details of ^1^Fe(CO)_4_ AX/EQ PDOS sampling, details of the geometry optimizations, relative energies of minima and transition states calculated using the B3LYP functional, details of the CASSCF CSF weights for the minima and transition states, and individual RDF plots of ^1^Fe(CO)_4_–OH and ^1^Fe(CO)_4_–CH (PDF)
