# Supplementary material for: Theoretical Investigation of Transient Species Following Photodissociation of Ironpentacarbonyl in Ethanol Solution
Source: Inorg Chem. 2024 May 28;63(23):10634–47. doi: 10.1021/acs.inorgchem.4c01100 (PMC11167646; doi:10.1021/acs.inorgchem.4c01100)
Supplement: Supplementary file 1 — ic4c01100_si_001.pdf [file ic4c01100_si_001.pdf]

Supporting Information for:

Theoretical Investigation of Transient Species following Photodissociation of Ironpentacarbonyl in Ethanol Solution.

Michael R. Coates,*,† Ambar Banerjee,†,‡,¶ Raphael M. Jay,‡ Philippe Wernet,‡ and Michael Odelius*,†

†*Department of Physics, Stockholm University, AlbaNova University Center, SE-106 91 Stockholm Sweden*

‡*Department of Physics and Astronomy, Uppsala University, Box 516, SE-751 20 Uppsala, Sweden*

¶*Current address: Research Institute for Sustainable Energy (RISE), TCG Centres for Research and Education in Science and Technology (TCG-CREST), Kolkata, 700091 India*

E-mail: michael.coates@fysik.su.se; odelius@fysik.su.se

Molecular orbital active space of $Fe(CO)_5$

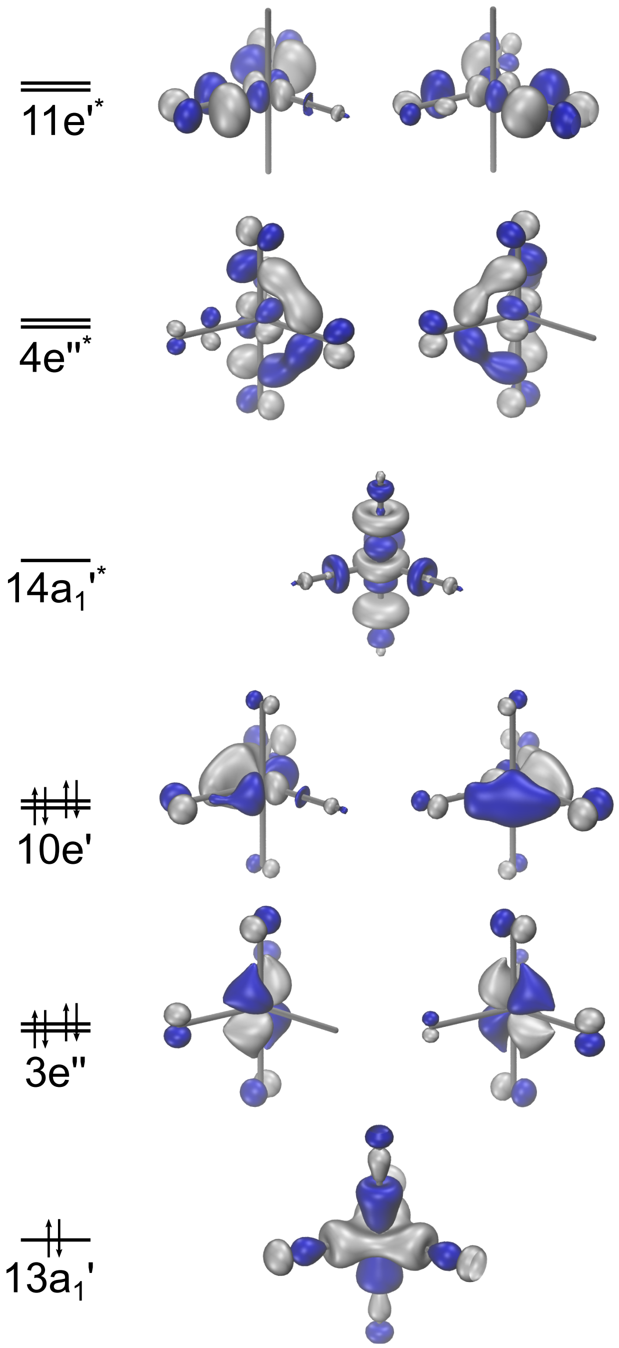

Figure S1: The CAS(10e,10o)/def2-TZVP orbitals containing the energetically relevant bonding and anti-bonding molecular orbitals of $Fe(CO)_5$.

Reactivity of singlet and triplet irontetracarbonyl

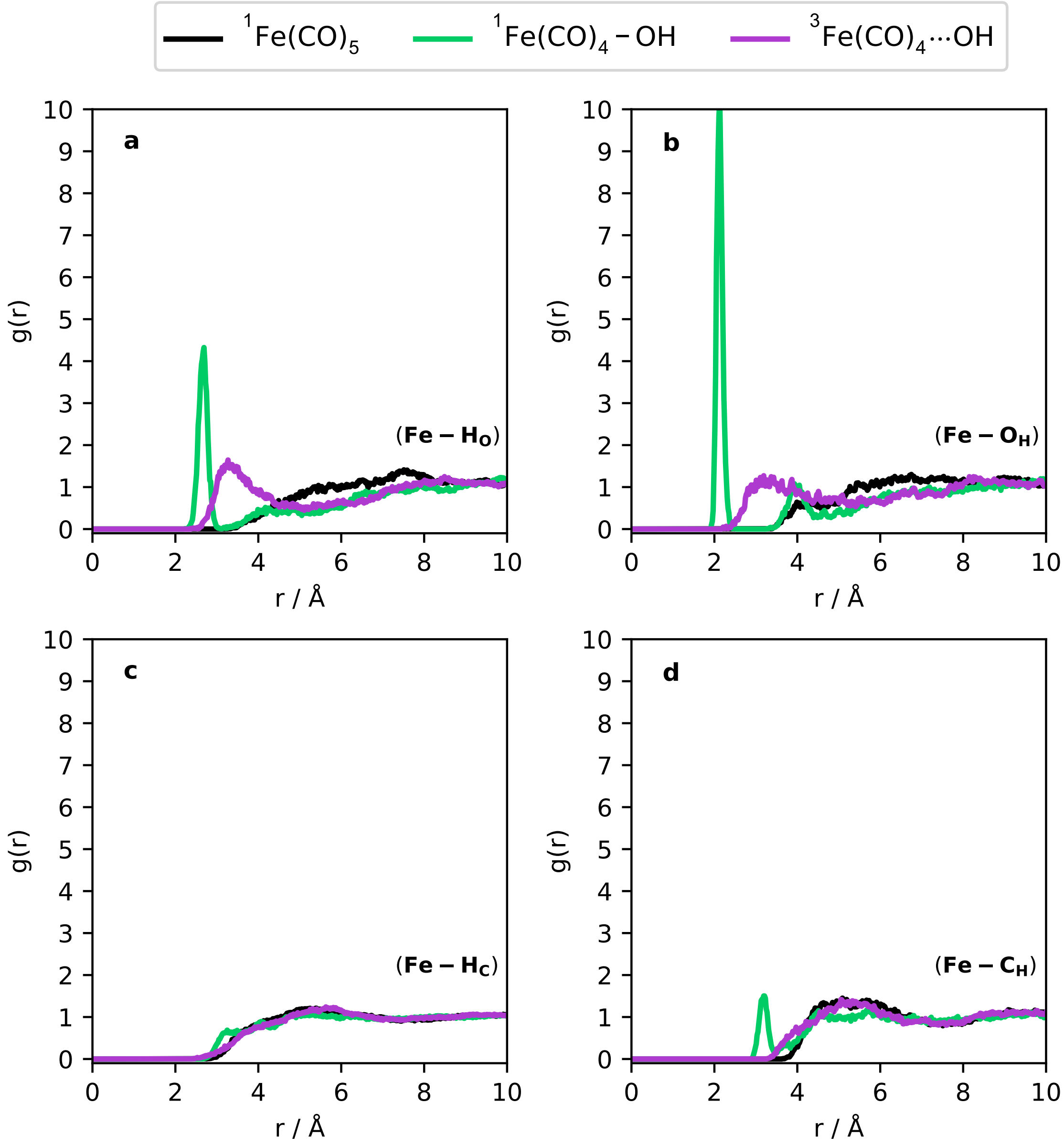

Figure S2: Radial distribution functions g(r) sampled from the $^1Fe(CO)_5$, $^1Fe(CO)_4$-OH and $^3Fe(CO)_4$ simulations. The coordination around the iron complexes are seen in g(r) for (a) Fe-H_O, (b) Fe-O_H, (c) Fe-H_C and (d) Fe-C_H. Results for $^1Fe(CO)_5$ are colored black, for $^1Fe(CO)_4$-OH are colored green and for $^3Fe(CO)_4$ are colored purple.

Angular fluctuations in solvated the $^1Fe(CO)_4$ complex

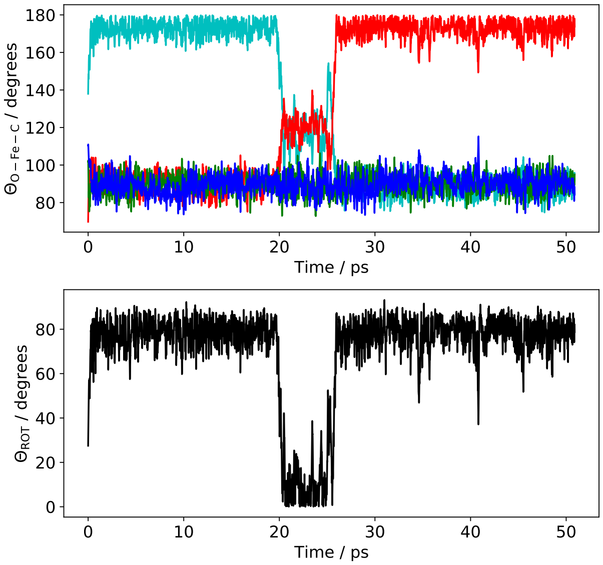

Figure S3: Top: the O-Fe-C angles ($\Theta_{\mathrm{O-Fe-C}}$) for the $^1\mathrm{Fe(CO)_4} - \mathrm{OH}$ simulation including the equilibrium (0-10 ps) and production (10-50 ps) runs. The indices of the axial and equatorial ethanol oxygen and carbonyl carbon atoms were identified at 0 ps and tracked for all times. The initial simulation of $^1\mathrm{Fe(CO)_4} - \mathrm{OH}$ AX is indicated by the three O-Fe-C angles of $\sim 90°$ and one O-Fe-C angle of $\sim 180°$. The region between 20 and ~26 ps containing two O-Fe-C angles of $\sim 90°$ and two O-Fe-C angles of $\sim 120°$ indicate the formation of $^1\mathrm{Fe(CO)_4} - \mathrm{OH}$ EQ. Following this region, the ligands exchange position and re-form the $^1\mathrm{Fe(CO)_4} - \mathrm{OH}$ AX complex. Bottom: The measure of the pseudo-rotation ($\Theta_{ROT} = \max(\theta_1 - \theta_2)$) defined as the difference between the two largest angles which tends to zero as the $^1\mathrm{Fe(CO)_4} - \mathrm{OH}$ EQ species is formed.

Triplet reactivity

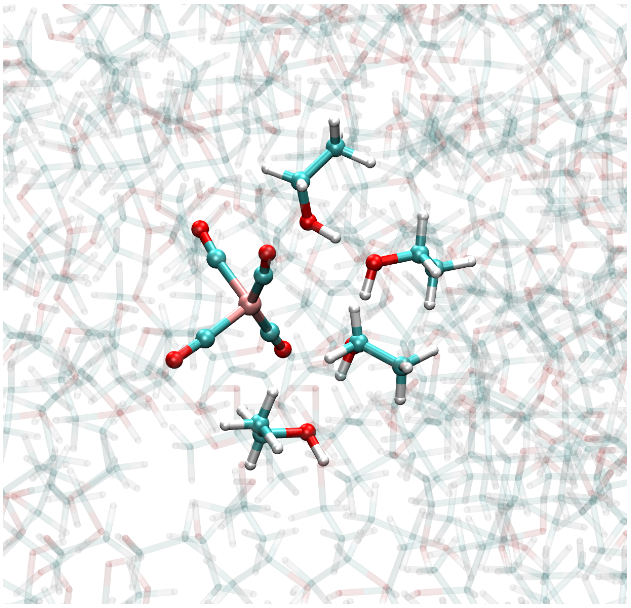

Figure S4: A qualitative picture of the solvation of $^3Fe(CO)_4$ indicates a small hydrogen bonding network of ethanol molecules around the weakly interacting metal complex.

Molecular orbitals of different complexes

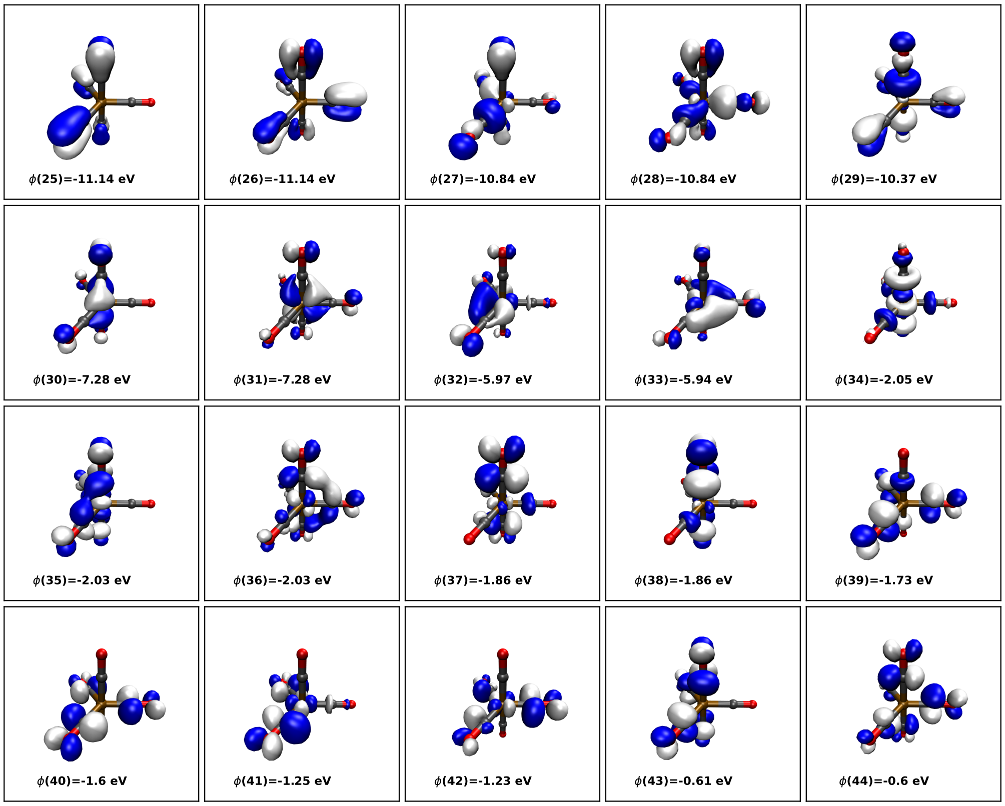

Figure S5: The energetically relevant valence molecular orbitals of ${}^{1}Fe(CO)_5$ plotted from the CP2K KS-DFT calculation using the BLYP functional and GTH pseudopotentials with the GTH-DZVP (Fe) and GTH-TZVP (C,O,H) basis sets. The HOMO is indicated by orbital label $\phi(33)$.

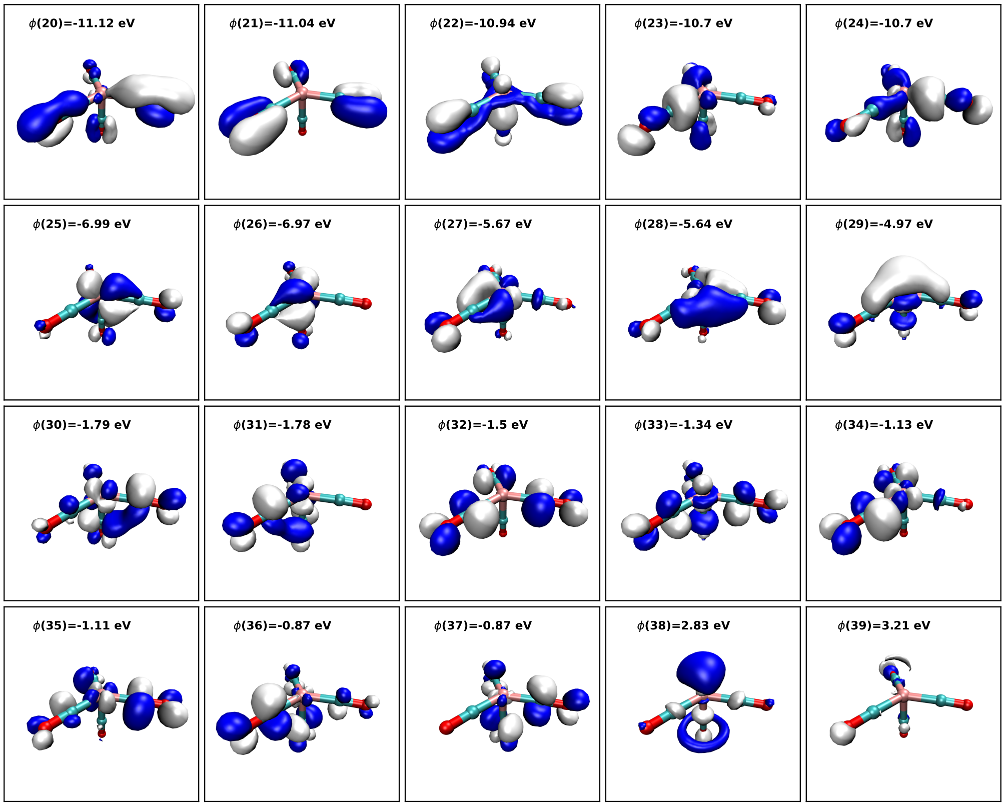

Figure S6: The energetically relevant valence molecular orbitals of $^{1}Fe(CO)_4$ AX plotted from the CP2K KS-DFT calculation using the BLYP functional and GTH pseudopotentials with the GTH-DZVP (Fe) and GTH-TZVP (C,O,H) basis sets. The HOMO is indicated by orbital label $\phi(28)$.

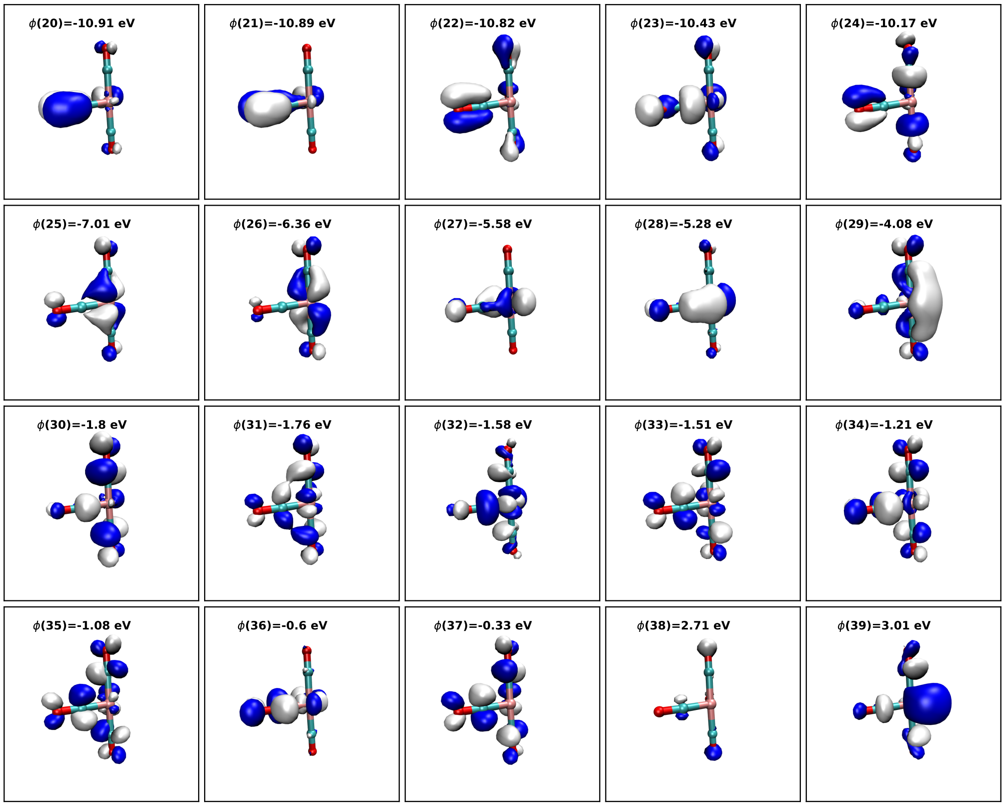

Figure S7: The energetically relevant valence molecular orbitals of ^{1}Fe(CO)$_4$ EQ plotted from the CP2K KS-DFT calculation using the BLYP functional and GTH pseudopotentials with the GTH-DZVP (Fe) and GTH-TZVP (C,O,H) basis sets. The HOMO is indicated by orbital label $\phi(28)$.

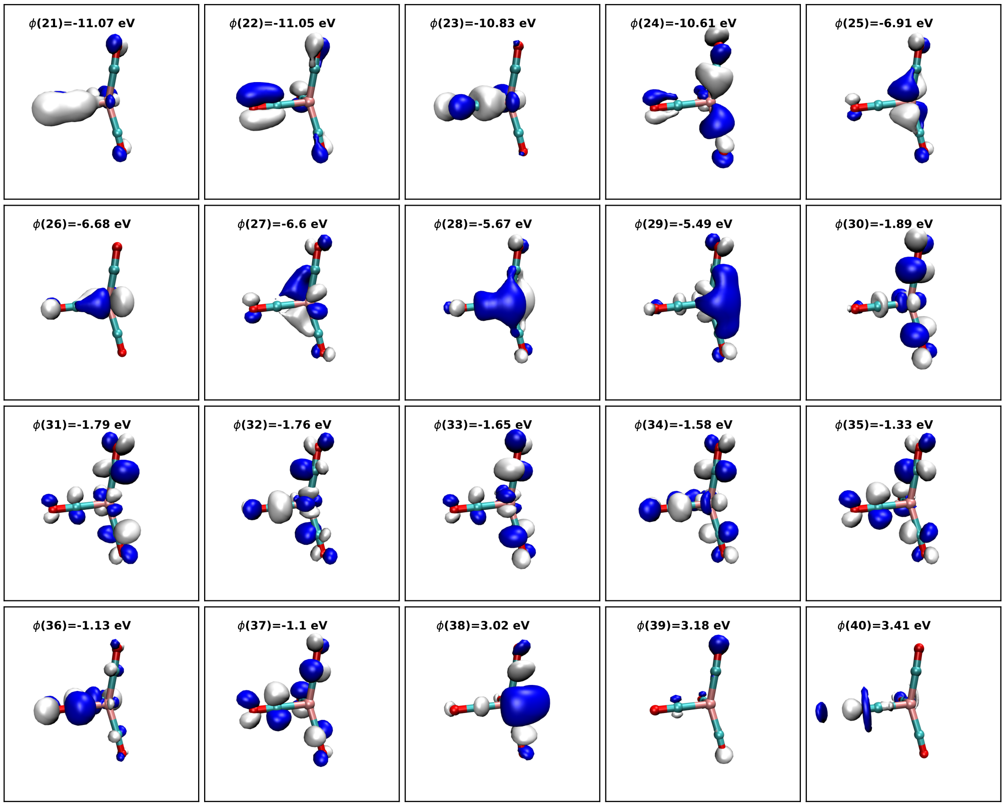

Figure S8: The energetically relevant valence molecular orbitals of $^3Fe(CO)_4$ (α-spin) plotted from the CP2K KS-DFT calculation using the BLYP functional and GTH pseudopotentials with the GTH-DZVP (Fe) and GTH-TZVP (C,O,H) basis sets. The HOMO is indicated by orbital label $\phi(29)$.

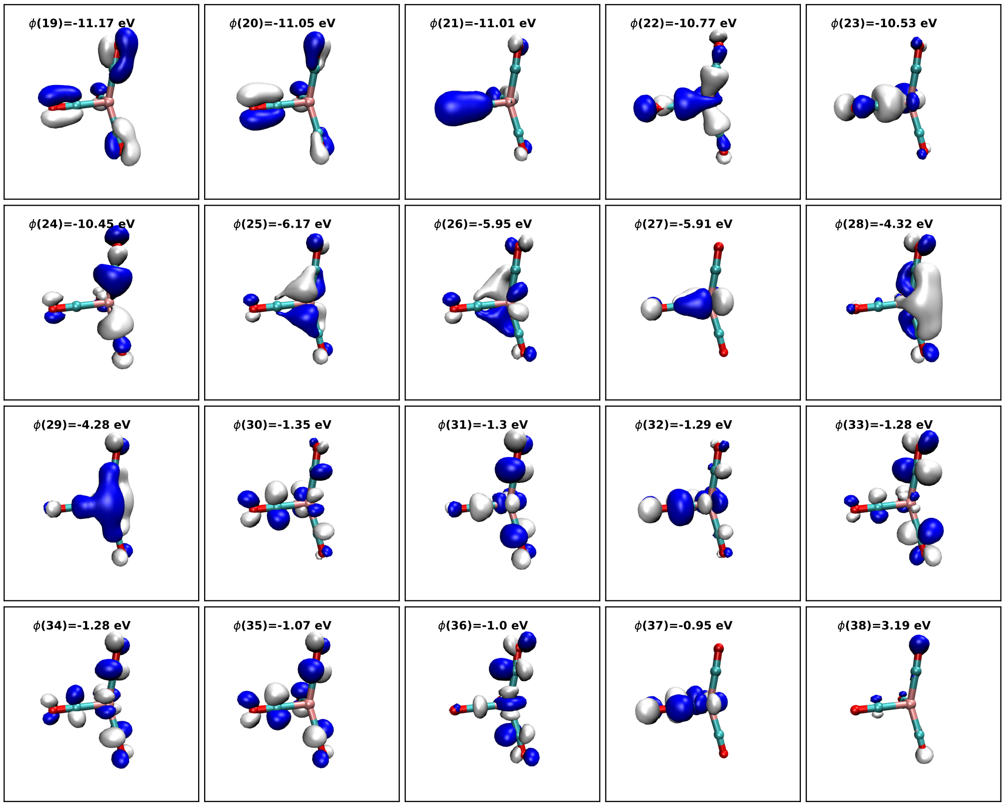

Figure S9: The energetically relevant valence molecular orbitals of $^{3}Fe(CO)_4$ (β-spin) plotted from the CP2K KS-DFT calculation using the BLYP functional and GTH pseudopotentials with the GTH-DZVP (Fe) and GTH-TZVP (C,O,H) basis sets. The HOMO is indicated by orbital label $\phi(27)$.

Chemical bonding for singlet irontetracarbonyl

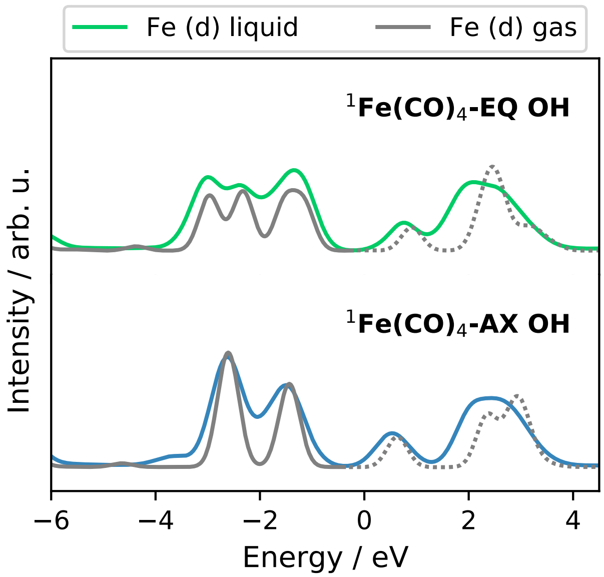

Figure S10: The average Fe (d) PDOS taken every 100 fs from the partitioned $^1Fe(CO)_4$-OH AIMD simulation into distinct regions of $^1Fe(CO)_4$-OH EQ (green) and $^1Fe(CO)_4$-OH AX (blue). The gas-phase Fe PDOS shown in grey are overlaid to indicate shifts in the peak positions and intensities.

The distinction between axial and equatorial signatures elucidated from the PDOS prompted a structural sampling to see how the orbital characters are embedded in the bulk liquid. In Fig. S10, we reproduce the $^1Fe(CO)_4$-OH AX sampling from Fig. 4 and include the averaged PDOS from the sampled $^1Fe(CO)_4$-OH EQ configurations in the $^1Fe(CO)_4$-OH AIMD simulation. Following the same sampling procedure defined in Sec. 3.1.1, we find that the gas-phase curves (shown in grey) map well onto the sampled PDOS for both kinds of coordination. This is indicative that the orbital characters of the molecular orbitals underlying the curves are preserved across different types of coordination (OH or CH) and in different environments.

Solvent coordination to singlet and triplet irontetracarbonyl

The structure library used throughout the main text and summarized in Tab. 1 contains a partially optimized structure, $[^1Fe(CO)_4 - HC_\alpha/HC_\beta]^\ddagger$ AX and one which contains a negligibly small imaginary frequency, $^3Fe(CO)_4 \cdots OH$.

For $[^1Fe(CO)_4 - HC_\alpha/HC_\beta]^\ddagger$ AX, the transition state was hard to obtain, therefore we constructed a 2D scan of the $^1Fe(CO)_4 - HC_\alpha$ ($Fe - H$ = 1.82 Å, 4.06 Å) and $^1Fe(CO)_4 - HC_\beta$ ($Fe - H$ = 1.77 Å, 3.18 Å) distances using ORCA 5.0.3[35] at the TPSSh/TZVP level of theory. An approximate transition state region of the potential energy surface was located and a minimum energy structure was obtained. Using this structure, the $C_\alpha H - Fe - HC_\beta$ angle was frozen to 57.1° and the structure was re-optimized in Gaussian[28] at the TPSSh/def2-TZVP + CPCM level of theory to yield an approximate transition state structure with only one imaginary frequency with a value of -170.56 cm^{-1}.

For $^3Fe(CO)_4 \cdots OH$, the structure was optimized without CPCM since the fragments were pushed further away from each other resulting in convergence problems. The structure that was obtained without CPCM contained one imaginary frequency with a value of -9.22 cm^{-1}. This imaginary mode corresponded to a small wagging of the two fragments, not involving the Fe-O distance. Nudging this structure away from the saddle point and optimization with CPCM resulted in a minimum 0.936 kcal mol^{-1} lower in energy at the TPSSh/def2-TZVP + CPCM level of theory. The structure that was obtained formed a bond with the hydroxyl proton and resulted in an Fe-O distance of 4.07 Å in contrast to the structure used in the paper which has an Fe-O distance of 3.29 Å. These structure are included in the structure library deposited Zenodo.

Table S1: Relative energies of minima and transition states, denoted with ‡, of the coordinations of an ethanol ligand with $^1Fe(CO)_4$ based on structures optimized at the TPSSh/def2-TZVP + CPCM(ethanol) level of theory. The uncoordinated $^1Fe(CO)_4$ AX, $^1Fe(CO)_4$ EQ and $^3Fe(CO)_4$ all include the energy based an optimized ethanol molecule. All structures are taken relative to the $^1Fe(CO)_4$ EQ + ethanol energy. The BLYP and B3LYP energies calculated using CP2K used a cell size of 30 Å to prevent interactions with all other periodic images without CPCM. All values are reported in kcal/mol. The double lines distinguish the geometries considered in Sec. 3.1 and those within the singlet coordination in Sec. 3.2

	BLYP (CP2K)	B3LYP (CP2K)
$^1Fe(CO)_4$-AX	0.00	0.00
$^3Fe(CO)_4$	-7.64	-7.11
$^1Fe(CO)_4$-OH AX	-27.83	-26.99
$^3Fe(CO)_4 \cdots OH^{\star}$	-9.44	-17.31
$^1Fe(CO)_4$-OH EQ	-24.91	-25.62
$^1Fe(CO)_4$-HC_α AX	-15.55	-7.40
$^1Fe(CO)_4$-HC_α EQ	-15.89	-12.76
$^1Fe(CO)_4$-HC_β AX	-12.18	-10.01
$^1Fe(CO)_4$-HC_β EQ	-14.14	-13.68
$[^1Fe(CO)_4\text{-OH}]^\ddagger$ AX/EQ	-23.35	-24.17
$[^1Fe(CO)_4\text{-}HC_\alpha]^\ddagger$ AX/EQ	-14.31	-8.48
$[^1Fe(CO)_4\text{-}HC_\beta]^\ddagger$ AX/EQ	-10.54	-5.00
$[^1Fe(CO)_4\text{-}HC_\alpha/OH]^\ddagger$ AX	-7.64	-2.24
$[^1Fe(CO)_4\text{-}HC_\alpha/OH]^\ddagger$ EQ	-12.22	-10.94
$[^1Fe(CO)_4\text{-}HC_\beta/OH]^\ddagger$ AX	-10.03	-1.20
$[^1Fe(CO)_4\text{-}HC_\beta/OH]^\ddagger$ EQ	-14.61	-9.15
$[^1Fe(CO)_4\text{-}HC_\alpha/HC_\beta]^\ddagger$ AX*	-7.24	3.68
$[^1Fe(CO)_4\text{-}HC_\alpha/HC_\beta]^\ddagger$ EQ	-11.08	-7.70

* See description above for details about these structures.

Table S2: The lowest three configuration state function (CSF) weights (c_0^2, c_1^2, c_2^2) based on structures optimized at the TPSSh/def2-TZVP + CPCM(ethanol) level of theory. The uncoordinated $^1Fe(CO)_4$ AX, $^1Fe(CO)_4$ EQ and $^3Fe(CO)_4$ all include the energy based an optimized ethanol molecule. All CSF weights are based on the a CAS(10e,10o)/def2-TZVP + CPCM(ethanol) reference calculation in ORCA. The double lines distinguish the geometries considered in Sec. 3.1 and those within the singlet coordination in Sec. 3.2

	c_0^2	c_1^2	c_2^2
^{1}Fe(CO)$_4$-AX	0.85757	0.01275	0.01081
^{3}Fe(CO)$_4$	0.86015	0.01554	0.01438
^{1}Fe(CO)$_4$-OH AX	0.85426	0.01293	0.01084
^{3}Fe(CO)$_4 \cdots$ OH*	0.86012	0.01545	0.01464
^{1}Fe(CO)$_4$-OH EQ	0.84456	0.01712	0.01424
^{1}Fe(CO)$_4$-HC$_\alpha$ AX	0.85731	0.01279	0.01040
^{1}Fe(CO)$_4$-HC$_\alpha$ EQ	0.85000	0.01546	0.01306
^{1}Fe(CO)$_4$-HC$_\beta$ AX	0.85618	0.01287	0.01074
^{1}Fe(CO)$_4$-HC$_\beta$ EQ	0.84906	0.01584	0.01333
[^{1}Fe(CO)$_4$-OH]‡ AX/EQ	0.85040	0.01442	0.01178
[^{1}Fe(CO)$_4$-HC$_\alpha$]‡ AX/EQ	0.85549	0.01203	0.01116
[^{1}Fe(CO)$_4$-HC$_\beta$]‡ AX/EQ	0.85547	0.01203	0.01138
[^{1}Fe(CO)$_4$-HC$_\alpha$/OH]‡ AX	0.85695	0.01285	0.01071
[^{1}Fe(CO)$_4$-HC$_\alpha$/OH]‡ EQ	0.84837	0.01556	0.01348
[^{1}Fe(CO)$_4$-HC$_\beta$/OH]‡ AX	0.85658	0.01278	0.01103
[^{1}Fe(CO)$_4$-HC$_\beta$/OH]‡ EQ	0.84833	0.01344	0.01335
[^{1}Fe(CO)$_4$-HC$_\alpha$/HC$_\beta$]‡ AX*	0.85731	0.01277	0.01080
[^{1}Fe(CO)$_4$-HC$_\alpha$/HC$_\beta$]‡ EQ	0.84967	0.01534	0.01291

* See description above for details about these structures.

Solvent coordination to singlet irontetracarbonyl

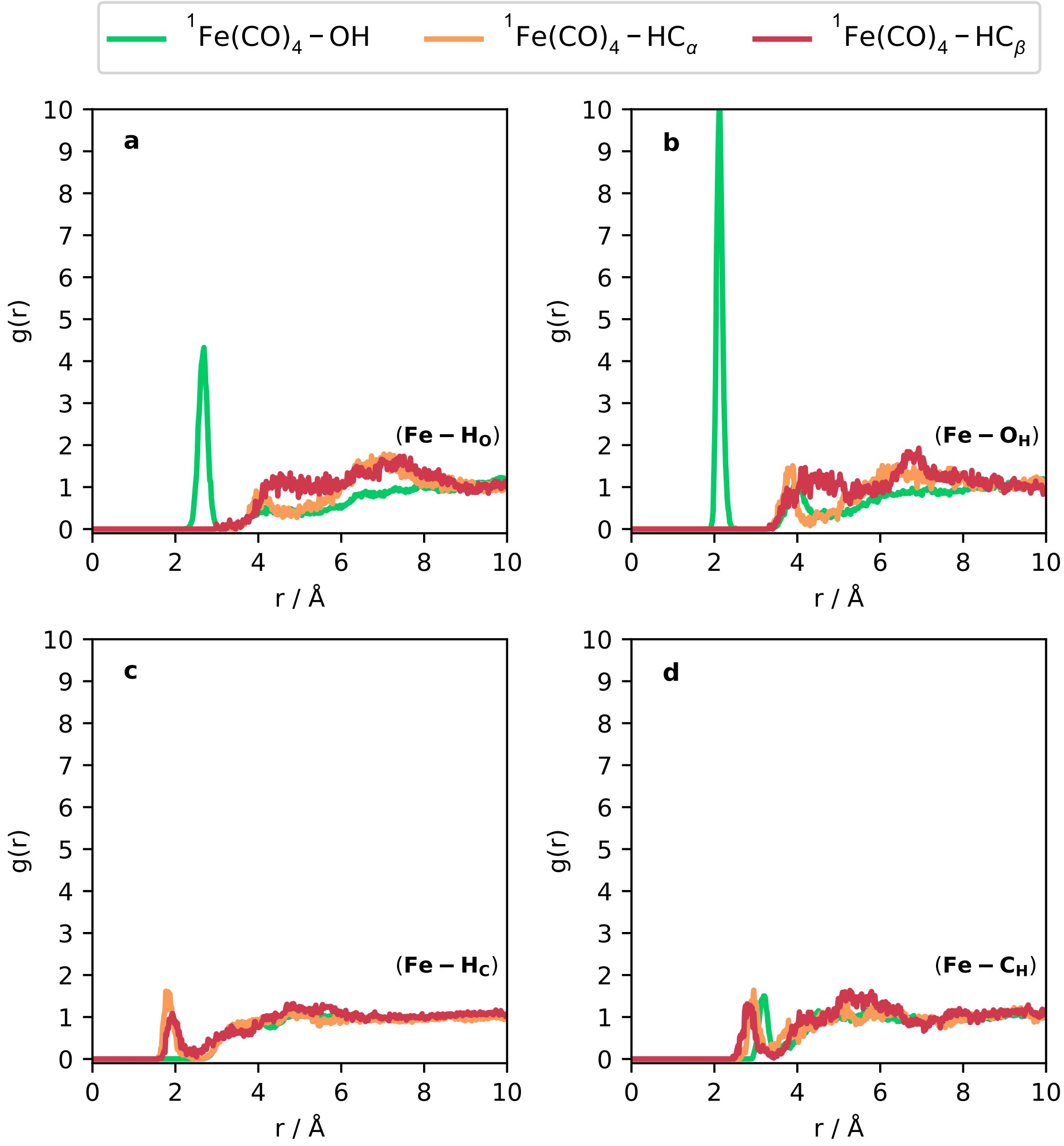

Figure S11: Radial distribution functions g(r) sampled from the $^1Fe(CO)_5$, $^1Fe(CO)_4$-OH and $^3Fe(CO)_4$ simulations. The coordination around the iron complexes are seen in g(r) for (a) Fe-H_O, (b) Fe-O_H, (c) Fe-H_C and (d) Fe-C_H. Results from $^1Fe(CO)_4 - OH$ are colored green, from $^1Fe(CO)_4$-HC_α are colored orange and from $^1Fe(CO)_4$-HC_β are colored red.
